# How to Make an Immune System and a Foreign Host Quickly Cohabit in Peace? The Challenge of Acute Graft-*Versus*-Host Disease Prevention After Allogeneic Hematopoietic Cell Transplantation

**DOI:** 10.3389/fimmu.2020.583564

**Published:** 2020-10-21

**Authors:** Benoît Vandenhove, Lorenzo Canti, Hélène Schoemans, Yves Beguin, Frédéric Baron, Carlos Graux, Tessa Kerre, Sophie Servais

**Affiliations:** ^1^ Laboratory of Hematology, GIGA-I3, GIGA Institute, University of Liège, Liège, Belgium; ^2^ Department of Clinical Hematology, University Hospitals Leuven, KU Leuven, Leuven, Belgium; ^3^ Department of Clinical Hematology, CHU of Liège, University of Liège, Liège, Belgium; ^4^ Department of Clinical Hematology, CHU UCL Namur (Godinne), Université Catholique de Louvain, Yvoir, Belgium; ^5^ Hematology Department, Ghent University Hospital, Ghent University, Ghent, Belgium

**Keywords:** allogeneic stem cell transplantation, acute graft-*versus*-host disease, T cells, alloreactivity, immune tolerance, tissue tolerance

## Abstract

Allogeneic hematopoietic cell transplantation (alloHCT) has been used as cellular immunotherapy against hematological cancers for more than six decades. Its therapeutic efficacy relies on the cytoreductive effects of the conditioning regimen but also on potent graft-*versus*-tumor (GVT) reactions mediated by donor-derived immune cells. However, beneficial GVT effects may be counterbalanced by acute GVHD (aGVHD), a systemic syndrome in which donor immune cells attack healthy tissues of the recipient, resulting in severe inflammatory lesions mainly of the skin, gut, and liver. Despite standard prophylaxis regimens, aGVHD still occurs in approximately 20–50% of alloHCT recipients and remains a leading cause of transplant-related mortality. Over the past two decades, advances in the understanding its pathophysiology have helped to redefine aGVHD reactions and clinical presentations as well as developing novel strategies to optimize its prevention. In this review, we provide a brief overview of current knowledge on aGVHD immunopathology and discuss current approaches and novel strategies being developed and evaluated in clinical trials for aGVHD prevention. Optimal prophylaxis of aGVHD would prevent the development of clinically significant aGVHD, while preserving sufficient immune responsiveness to maintain beneficial GVT effects and immune defenses against pathogens.

## Introduction

For almost 6 decades, allogeneic hematopoietic cell transplantation (alloHCT) has been the cornerstone of poor risk hematological cancer therapy. Although novel sophisticated cellular therapies (such as those with CAR T cells) have emerged and appear to be occupying a growing place in the modern therapeutic arsenal in hematology, their long-term effects on disease control and survival are still unclear. Therefore, alloHCT still remains standard of care in a variety of high risk hematological disorders, often offering the only curative option for these diseases (1). The persisting major role of alloHCT in current medicine is documented by the constant increase in the annual number of stem cell transplants performed worldwide, with >19,000 alloHCT procedures in Europe and associated countries in 2018 (701 centers in 50 countries) (2). In adults, the most frequent indications for alloHCT remain acute leukemia (more than 50% of all alloHCT), followed by myelodysplastic syndromes and non-Hodgkin lymphoma (2). In addition, an acceptable donor can currently be found for almost all patients, mainly due to the recent development of innovative platforms for alloHCT with HLA-haploidentical family donors (mismatched for one of the two HLA haplotypes).

The therapeutic efficacy of alloHCT against hematological cancers relies on the cytoreductive effects of the conditioning regimen but also (and mainly) on potent graft-*versus*-tumor (GVT) reactions, defined as immune-mediated reactions by donor cells against tumor cells. However, beneficial GVT effects may be counterbalanced by acute GVHD (aGVHD), a systemic syndrome in which donor immune cells attack healthy tissues of the recipient, resulting in severe inflammatory lesions mainly of the skin, gut and liver. Despite more than 6 decades of preclinical and clinical researches, the immunological requirements necessary to achieve GVT effects without promoting aGVHD have not been fully established.

Despite standard prophylaxis regimens, aGVHD occurs in approximately 20–50% of transplanted patients and is a major cause of treatment failure and mortality after alloHCT. Therefore, the prevention of aGVHD after alloHCT represents an unmet medical need in the modern era of cancer immunotherapy and research must continue in this field. Here, we provide a brief overview of criteria for aGVHD diagnosis and grading as well as current knowledge on aGVHD immunopathology. Then, we discuss current approaches and novel strategies being developed and evaluated in clinical trials for aGVHD prevention.

## What is aGVHD? The Clinical Point of View

GVHD is separated into two syndromes, historically defined according to the time frame of occurrence of symptoms: acute GVHD (aGVHD) occurring within the first 100 days after transplantation and chronic GVHD (cGVHD) developing thereafter. Although simple, this classification based only on empirical observations and did not rely on actual biological or clinical bases. More recent classification systems have emphasized differentiating a- and cGVHD based on pathophysiological mechanisms and clinical manifestations (3, 4).

In 2018, a consortium of GVHD experts from the European Society for Blood and Marrow Transplantation (EBMT), the National Institutes of Health (NIH) and the Center for International Blood and Marrow Transplant Research (CIBMTR) reviewed the terminology and guidelines for GVHD diagnosis and scoring (5). Clinically, aGVHD typically presents with inflammatory lesions, the three main organs involved being: the skin (erythematous and pruriginous maculopapular skin rash), the gastro-intestinal (GI) tract (nausea, vomiting, and anorexia with weight loss in the upper tract; and/or watery or bloody diarrhea, crampy abdominal pain and/or ileus in the lower tract), and the liver (cholestasis with hyperbilirubinemia) (5, 6). Typical aGVHD is defined by the presence of these exclusive inflammatory manifestations, without any other sign consistent with cGVHD. Ideally, the diagnosis of aGVHD should be confirmed by positive histological findings, but this is not formally required (5). AGVHD can be categorized as “*classic aGVHD”* in the setting of typical aGVHD manifestations occurring less than 100 days after alloHCT or donor lymphocyte infusion (DLI), and “*late, recurrent or persistent aGVHD”* in patients with typical aGVHD signs experienced later than 100 days after alloHCT/DLI (5, 7).

Grading aGVHD is essential because it is predictive of non-relapse mortality and it guides therapeutic management. Several scoring systems have been developed during the past decades, including the original Glucksberg classification (first established in the 1970s), the “Modified Glucksberg” or “Keystone”, the IBMTR and the “MAGIC” scoring systems (8–11). Each of them proposes a 4-grade scale, integrating the individual stage of each target organ (skin, GI tract, and liver), with or without the general *Performance Status*. Recently, the EBMT−NIH−CIBMTR Task Force Consortium recommended the MAGIC criteria as the most accurate and detailed clinical criteria for diagnosis and grading the severity of aGVHD (5). A web-application has also been developed based on this position statement (*eGvHDApp; https://www.uzleuven.be/egvhd*) and has been found to be helpful in improving aGVHD and cGVHD scoring consistency and compliance with guidelines (12, 13).

In addition to the typical manifestations of aGVHD in the skin, GI, and liver, there is accumulated evidence that aGVHD may also affect other tissues, including the cellular niches in the bone marrow (BM), thymus and secondary lymphoid organs (14). Although lesions in these organs are hardly clinically detectable, they can severely impact outcome by impairing hematopoiesis, compromising T- and B-cell reconstitution and predisposing to the development of subsequent cGVHD (15). It has also been suggested that aGVHD can cause damages to the endovascular endothelium and can be the trigger of endothelitis-related complications after alloHCT, such as transplant-associated microangiopathy, diffuse alveolar hemorrhage, idiopathic pneumonia syndrome (16). Finally, over the past decade, experimental data (17, 18) and clinical case reports (19) have gradually accumulated suggesting that the central nervous system may also be a potential target of aGVHD. Although they are not considered in current standard aGVHD diagnosis criteria and grading systems, alloreactive lesions to these tissues can be associated with significant morbidity.

Despite conventional prophylactic measures, it is estimated that 20–50% of transplanted patients develop clinically significant grades II–IV aGVHD after alloHCT. Known risk factors include the stem cell source (G-SCF mobilized peripheral blood stem cells, PBSC), the donor type (unrelated, female donor for a male recipient), the degree of donor/recipient HLA-mismatch, the intensity of the conditioning regimen (myeloablative regimen), the occurrence of severe infections during the peri-transplant period and administration of DLI (7, 20, 21).

The standard first-line of treatment for grades II–IV aGVHD is high-dose systemic corticosteroids. However, aGVHD fails to respond to steroids in approximately 30–50% of patients (the risk increasing with increasing grade), therefore requiring subsequent lines of immunosuppressive therapies (22, 23). Outcomes of patients with steroid refractory aGVHD have been dismal (up to 60–85% of non-relapse mortality at 2 years), partly due to aGVHD by itself, but also to cumulative toxicity and increasing susceptibility to infections and relapse incurred with additional immunosuppressive therapy (22, 24). Hopefully, research is constantly developing in the field and two recent large phase III studies have provided significant benefit in efficacy outcomes with two novel strategies for the treatment of steroid refractory aGVHD. First, Socie et al. reported better long-term overall survival with inolimomab (an anti-CD25 monoclonal antibody) in comparison with anti-T cell globulin (25). The second phase III study demonstrated higher response rate with ruxolitinib (a JAK 1–2 inhibitor) compared to the investigator's therapy of choice (26). Nevertheless, aGVHD remains a severe complication and one of the major cause of early post-transplant mortality (27).

## What is aGVHD? The Immunological Point of View

Despite significant improvements in the field over the past 20 years, the complex immunobiology of aGVHD still remains only partially elucidated. Here, we present a simplified overview of the main basic immunological concepts on aGVHD biology, with the aim of providing readers with some clues for understanding the rationale of both current and emerging preventive approaches. For more detailed information about aGVHD pathophysiology, readers are referred to several outstanding reviews (6, 28–30).

### Donor T Cells as Drivers, Amplificators, and Effectors of aGVHD Responses

AGVHD after alloHCT mainly results from donor T-cell alloreactivity against the recipient's tissues, as evidenced by the low incidence of GVHD observed in patients transplanted with a T-cell depleted allograft (31). After alloHCT, transferred donor T cells are able to recognize structurally dissimilar allogeneic peptide/HLA complexes in the recipient, reacting against either polymorphic HLA molecules (in case of alloHCT with HLA-mismatched donor/recipient pair) and/or peptides (minor histocompatibility antigens) presented by either shared or dissimilar HLA molecules (in the setting of alloHCT with HLA-matched or mismatched donor/recipient pair, respectively) (32–34).

**Figure 1:**
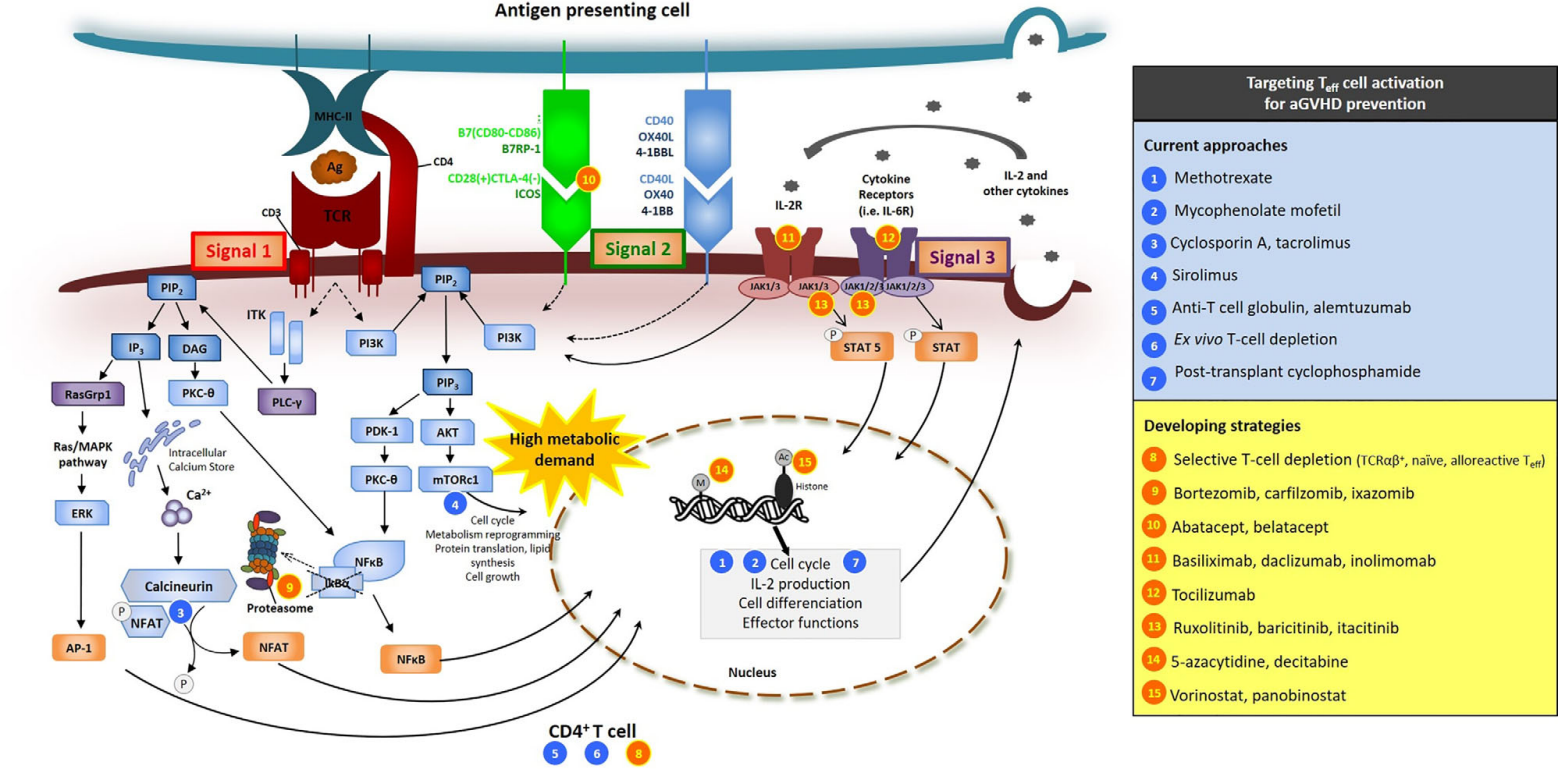
Signals 1, 2, 3 of T-cell activation and sites of action of several molecules used or tested in clinical trials for aGVHD prevention. Sites of action of current approaches (blues circles) and developing strategies (orange circles) are presented. Beyond their effects on T_eff_, several of these molecules have also effects on other cell types (see text). [adapted from (35)].

In general, three types of signals are required to generate full alloreactive T-cell responses after alloHCT (**Figure 1**) (6, 28–30, 35). The first triggering event that makes a donor T cell alloreactive is the activation of its TCR by the peptide/HLA complex (signal 1). TCR engagement leads in the activation of a series of intracellular downstream signaling pathways that ultimately result in the nuclear translocation of key transcription factors such as *nuclear factor-kappa B* (NF-*κ*B), *Adaptor-related Protein complex 1* (AP1), and *nuclear factor of activated T cell* (NFAT), whose coordinated activity orchestrates the complete activation of the T cell, its proliferation and its synthesis of cytokines and cytokine receptors, such as IL-2 and CD25 (the *α* subunit of the high affinity *αβγ* forms the IL-2 receptor) (36). Besides the basic biology, the blockade of one of these TCR-downstream signaling pathways, namely the NFAT calcium/calcineurin-dependent transduction pathway, was one of the first strategies explored to repress alloreactive T-cell activation after alloHCT in pioneered preclinical and clinical studies (37) and is still currently universally used as a standard approach for aGVHD prophylaxis (see below). Inhibition of the NF-*κ*B pathway was also demonstrated to be efficacious for reducing proliferation, survival, cytotoxic functions and production of cytokines in alloreactive T cells during aGVHD (38–40).

Along with TCR activation, additional positive costimulation (*signal 2*) is required to allow complete T-cell activation and avoid anergy or apoptosis (41). Multiple T-cell positive costimulatory molecules have been identified to play role in aGVHD, such as CD28, *inducible co-stimulator* (ICOS), OX40, and 4-1BB [nicely reviewed in (41, 42)] (**Figure 1**). Their cognate ligands [namely B7 ligands (CD86 or CD80), *B7-related protein-1* (B7RP-1), OX40L and 4-1BBL, respectively] are highly expressed at the surface of mature antigen presenting cells (APCs). Among all of the T-cell costimulatory receptors, the most extensively studied is CD28, which is constitutively expressed at the surface of naive T cells. Another B7 receptor, induced with T-cell activation, is *cytotoxic T-lymphocyte-associated protein 4* (CTLA-4) that has similar structure to CD28 and acts as a competitor for CD80 and CD86 ligation, resulting in dowregulation of T-cell responses. Blockade of CD28/B7 interactions has been shown to attenuate alloreactive T-cell activation, induce tolerance to host alloantigens and to reduce aGVHD in *in vitro* studies and animal models of alloHCT (43–46). One of these approaches consists in using fusion proteins of the Fc region of human immunoglobulin with the extracellular domain of CTLA4 (CTLA4-Ig) (43, 45) and is tested for aGVHD prevention in clinical trials (see below).

The third signal for sustained T-cell activation, acquisition of effector functions and survival is provided by cytokines [*signal 3*, nicely reviewed in the context of aGVHD in (42, 47)]. Among all, IL-2 is a key cytokine for alloreactive T-cell proliferation and survival. Produced by activated T cells, IL-2 acts through paracrine and autocrine signaling, further providing a self-activation loop. Among others, IL-2 receptor intracellular signaling in T cells include JAK (janus kinase)/STAT (*signal transducers and activators of transcription*) pathways (namely JAK1 and JAK3/STAT5 in particular) (**Figure 1**) (48, 49). JAK1/2 pathways are also involved in signal transduction downstream the receptors of multiple other cytokines (such as IL-6, IL-12, ...) and play major role in T-cell proliferation, polarization, and metabolic reprogramming (49). Pharmacological inhibition of JAK1/2 pathways was demonstrated to reduce aGVHD in preclinical models (50) and to be efficacious for the treatment of patients with steroid refractory-aGVHD (see above, *INTRODUCTION*) (26). Mechanisms of JAK1/2 inhibition on aGVHD reactions include at least decreased Th1 and Th17 differentiation, but also broad anti-inflammatory and immunosuppressive effects on multiple immune cell types [reviewed in (50)]. It is commonly accepted that pathogenic Th1 and Th17 cells as well as their polarizing cytokines [IL-12 and interferon gamma (IFN*γ*); IL-6, IL-1*β*, IL-21 and IL-23, respectively] play important role during aGVHD (42, 47, 51). Among all cytokines, IL-6 is the hallmark of pro-inflammatory cytokines and increased systemic IL-6 levels were reported in patients early after alloHCT (52, 53). IL-6 signaling in donor T cells is critical for the polarization of donor naive T cells towards Th17/Tc17, but IL-6 also exerts many other effects (such as several on DC and regulatory cells) (54, 55).

*Mechanistic/mammalian target of rapamycin* (mTOR) is another key signaling kinase in T cells that integrate an array of activating signals (including the three aforementioned signals of T-cell activation) and environmental cues to regulate cell survival, growth, proliferation, differentiation, and metabolism (56). Inhibition of mTOR Complex 1 (mTORC1) has demonstrated efficacy against aGVHD in preclinical models (56–58) and has been explored as GVHD prevention in clinical trials for several years (see below).

Over the past decade, it has become increasingly clear that metabolic reprogramming of the T cell is required to enable the transition from a naive T cell to a proliferative and differentiated T cell that will drive immune effector functions and mediate aGVHD. Studies have reported that effector T cells use multiple metabolic pathways (glycolysis, oxidative phosphorylation, fatty acid oxidation, glutaminolysis) to keep the pace with high energy demands during aGVHD, (59, 60). Furthermore, the metabolic demand of different T cell subsets is likely not identical.

A key event in the initiation phase of aGVHD is the interaction of CD4^+^ and CD8^+^ donor T cells with activated APCs (*via* cross-presentation for the latter) that provide the three aforementioned signals. During the initiation phase of aGVHD, most of the APCs are host-derived hematopoietic APCs and host non-hematopoietic APCs (intestinal epithelial cells, keratinocytes, myofibroblasts...) (61, 62). By expressing pattern recognition receptors (PRR) such as Toll-like (TLR) and nucleotide oligomerization domain (NOD)-like receptors, innate immune cells and some epithelial cells are able to detect danger signals such as sterile DAMP (*damage-associated molecular pattern* molecules, which are released from dying cells or disrupted extracellular matrix) and PAMP (*pathogen-associated molecular pattern* molecules, which can be released from invasive bacteria, fungi or viruses at the epithelial surfaces). After alloHCT, an increased number of DAMP and PAMP molecules can be released as a consequence of cytotoxic conditioning regimen or aGVHD [reviewed in (63)]. After alloHCT, several studies have demonstrated that host exposure to gut microbial flora and PAMPs due to disrupted intestinal barrier can be an important initiating event in aGVHD reactions (64–67). Mechanisms include the recruitment and activation of host neutrophils (which further contribute to tissue damage and inflammation) as well as inflammatory macrophages, dendritic cells and non hematopietic APCs (which further prime T cells) (61, 67–69).

Beyond T-cell activation and clonal expansion, T-cell chemotaxis towards secondary lymphoid organs and target tissues are also important in aGVHD immunobiology [nicely reviewed in (70)]. For example, among the so-called "homing receptors", the chemokine-receptor CCR7 and the L-selectin (CD62L) are expressed at the surface of naive and central memory T cells and direct them to secondary lymphoid organs in which they can be primed and activated by professional APCs. This raises the hypothesis that T cells may contribute differently to aGVHD according to their differentiation status, with naive CD4^+^ T cells being more prone to cause aGVHD than (late) effector memory CD4^+^ T cells (71, 72). In addition, T-cell migration towards GVHD target organs is also crucial to cause aGVHD. Namely, the chemokine receptor CCR5 is involved in T-cell migration towards lymph nodes, the GI tract and the liver. Hence, CCR5-chemotaxis blockade was reported to limit aGVHD in some murine models (73, 74). Integrins also participate in T-cell migration to target organs and the specific tissue expression of some of them may make their study interesting in the context of aGVHD. Several mouse studies have indeed suggested that *α*4*β*7 integrin on donor T cells was important for T-cell migration into gut-associated lymphoid tissues and for the development of GI aGVHD (75, 76).

After being primed by APCs in secondary lymphoid organs, activated and differentiated donor T cells migrate to target organs where they generate effector T cell (T_eff_) responses (effector phase of aGVHD). Cytotoxic T cells can cause direct target tissue cell death *via* diverse cytolytic pathways that involve the release of granzyme B and perforin and the expression of members of the *tumor necrosis factor* (TNF) family (including FasL). Immune activation and tissue lesions lead to a cytokine storm that further recruits multiple cellular effectors (*e.g.* other T cells, neutrophils, and activated macrophages) and brings molecular effectors (*e.g.* TNF-*α*, IFN-*γ*, complement molecules, reactive oxygen species, ...), further intensifying tissue lesions and inflammatory responses (amplification phase) and thus leading to sustained aGVHD reactions and severe end-organ damages.

### Mechanisms Establishing Immune Cell and Tissue Tolerance During aGVHD

As mentioned above, T_eff_ cell activation and proliferation are negatively regulated by co-inhibitory signals. In addition to these T-cell intrinsic pathways, peripheral immune tolerance can also be achieved by the intervention of several anti-inflammatory molecules as well as tolerogenic cells. In the context of aGVHD, all of these components can help restraining the destructive machinery of immune cell and limiting tissue damages.

Numerous investigations have focused on regulatory T cells (T_regs_), which can exert multiple tolerogenic and anti-inflammatory effects [nicely reviewed in (77–79)]. T_regs_ are characterized by the expression of the master *forkhead box protein 3* transcription factor (FoxP3) and their constitutive expression of the surface receptor CD25, the high affinity IL-2R *α*-chain (in contrast to Teff in which CD25 expression starts upon the TCR activation) (80). Hence, in steady-state conditions (low dose of IL-2), T_regs_ capture all the IL-2 molecules in the milieu, therefore quenching spurious activation of T_eff_. There are several types of CD4^+^ T_reg_: (1) "*natural thymus-derived T_reg_"* (nT_reg or_ tT_reg_), generated from lymphoid precursors in the thymus; and (2) "peripheral T_reg_" (pT_reg_), derived from the differentiation of conventional naive T cells in secondary lymphoid organs in the context of low-dose or tolerogenic antigen exposure and upon IL-10 and TGF-*β* stimulation. pT_reg_ can also be generated *in vitro* and in this case are referred as “*induced T_reg_*” (iT_reg_). Interestingly, preclinical studies in mice have shown that co-transplanting high doses of CD4^+^ iT_reg_ or infusing fewer freshly isolated T_reg_ from donor peripheral blood (likely containing a mixture of t- and pT_reg_) several days prior to alloHCT in lymphopenic conditions was effective for mitigating allogeneic and human xenogeneic GVHD (81, 82). However, one issue with T_reg_ adoptive transfer could be their phenotypic and functional instability in the context of prolonged inflammation (such as during aGVHD), causing them to lose their immunosuppressive properties and even acquire pro- inflammatory functions. Such observations were made in mice (83, 84) but also with human T_reg_ in the context of xenogeneic GVHD (85).In comparison to tT_reg_, the expression of FoxP3 is more unstable in iT_reg,_ since they lack the locked-in gene expression signature of transcription factors implicated in FoxP3 activity stabilization. Specifically, hypermethylation of FoxP3 gene/promoter in iT_reg_ was reported to destabilize their phenotype (86). By contrast, phenotypic and functional stabilization of T_reg_ cells has been demonstrated with hypomethylating agents in a model of xenogeneic GVHD (87).

Type 1 regulatory T cells (Tr1) are another subset of suppressive peripheral T cells, still suppressing immune response similarly to t- and pT_reg_ but characteristically lacking CD25 and Foxp3 lineage marker expression (88). Although if this subpopulation has been only partly unraveled so far, Tr1-like cells are being considered more and more important for immune response homeostasis. Similarly to iT_reg_, Tr1-like cells can be induced *in vitro* (88, 89), and a recent preclinical co-transfer study has shown promising results for suppressing GVHD (89).

Other cell types that have been reported to exert immunoregulatory properties during aGVHD, include invariant natural killer T cells (iNKT), natural killer cells (NK), innate lymphoid cells (ILC), tolerogenic dendritic cells, various myeloid suppressor populations of hematopoietic [*e.g*., myeloid-derived suppressor cells {MDSCs}, CD34+ regulatory monocytes] and stromal origin [*e.g*., mesenchymal stromal cells (MSCs)] (90–92). In particular, iNKT cells are under increased investigation, owing to their reported suppressive activity against GVHD in preclinical models (93, 94).

In addition to these tolerogenic immune cell subtypes, other non-immune cells and components of the damaged organs can also reveal protective properties in the context of aggression, through several mechanisms including the up-regulation of anti-inflammatory surface receptors, release of tolerogenic soluble factors and activation of repairing mechanisms (a concept known as “tissue-tolerance”). This concept has been described in recent nice articles (95, 96). Among others, IL-22, keratinocyte growth factor (KGF), R-spondin-1 (R-Spo1) and glucagon-like peptide 2 (GLP-2) were reported to be protective against GI manifestations of aGVHD (*e.g.* by preserving and/or enhancing the regeneration of intestinal epithelial cells, intestinal stem cells and/or Paneth cells) (97–101). Paneth cell secretion of antimicrobial peptides (*e.g.* *α*-defensin) is also critical for maintaining the GI microbial ecosystem (97).

There is also growing evidence that the commensal microbiota at mucosal and cutaneous surfaces plays important role in tissue homeostasis and immune tolerance after alloHCT. This concept has been particularly studied at the intestinal interface [nicely reviewed in (102)]. It was recently reported that the bacterial and viral gut microbiota is altered (with loss of diversity and dominance of some taxa) after alloHCT and that such dysbiosis may be associated with aGVHD outcomes (103–107). Regarding bacteria, low intestinal abundance of gut commensals belonging to the *Lactobacillales*, *Clostridiales* and *Blautia* genus was reported to be associated with and increased incidence of lethal aGVHD and poor survival (104, 108). Consistent with this, increased risk of aGVHD-related death was also reported with the use of some anti-anaerobic or broad-spectrum antibiotics in mice and in patients (104, 109–111). However, most of the aforementioned studies were based on associations, and the causations as well as the precise mechanisms of how the microbiota can influence immune and tissue tolerance post-allo-HCT remain to be determined. Recent data suggested that an important way could be through microbiota-derived metabolites (112, 113). A recent elegant work has indeed highlighted significant variations in microbiota-derived metabolites (especially aryl hydrocarbon receptor ligands, bile acids and plasmalogens) at the onset of aGVHD in patients (114). A significant reduction in fecal levels of butyrate [a short-chain fatty acid (SCFA) generated by the fermentation of non-digestible carbohydrates by certain anaerobic commensal bacteria] in patients after alloHCT was also alloHCT reported by another group (114). Interestingly, in a mouse model, restoring butyrate levels, either by direct administration of butyrate or by changing the composition of intestinal microbiota towards an increase in butyrogenic bacteria (*e.g.* selected strains of *Clostridia*) mitigated aGVHD and improved survival (115). Understanding the precise effects of all these metabolites on host tissues and immunity is the subject of intense current research, with some data already suggesting various potential roles in enhancing the trophicity and regenerative properties of the intestinal epithelium as well as in modulating innate and adaptive immune responses (102, 112, 113). Overall, these findings highlight the likely major role of the microbiome-metabolome axis in aGVHD, which may offer potential new targeted strategies to explore for improving aGVHD prophylaxis or treatment.

## How to Prevent aGVHD After alloHCT? When the Clinician Meets the Immunologist

### Conventional Strategies for aGVHD Prevention

Currently, there is no standardized aGVHD preventive approach. However, the backbone of most conventional prophylactic regimens is based on T-cell immunosuppression, by the pharmacological inhibition of their clonal expansion and activation and/or by their direct depletion (116). Here, we provide a short overview of the current standard regimens for aGVHD prevention and briefly describe their biological rationale. For detailed clinical considerations, readers are referred to the recently published 2019 EBMT consensus recommendations for aGVHD prophylaxis and treatment (117).

Since the mid-1980s (37), the most commonly adopted GVHD prophylaxis regimens among patients given alloHCT with BM or PBSC from HLA-matched sibling or unrelated donor consist in the combination of an anti-metabolite [either short course of methotrexate (MTX) or mycophenolate mofetil (MMF)] with a calcineurin inhibitor [CNI, either cyclosporin A (CSA) or tacrolimus (FK506, tacro)]. The former (MTX or MMF) delete proliferating T cells, while the second (CNI) blocks TCR-induced T-cell activation (*signal 1*) by interfering with NFAT nuclear translocation thereby reducing transcription of IL-2 (**Figure 1**).

Several other alternative regimens have also been explored with the aim of improving the control of aGVHD and/or reducing drug toxicity. Among them, administration of mTOR inhibitors [of which sirolimus (siro) is the most widely studied molecule] has been tested for several years (**Figure 1**). Unlike CNIs which, by reducing IL-2 production, limit T_eff_ activation but with a concomitant negative impact on IL-2-dependent T_reg_, inhibition of the mTOR signaling pathway precludes the activation of T_eff_ while preserving T_reg_ activity (which are less dependent on the mTOR/Akt pathway) (58, 118). Several randomized phase III trials have addressed the effects of siro either as a substitution of MTX (tacro + siro *vs.* tacro + MTX) in myeloablative TBI-based alloHCT (119) or in addition to the standard prophylaxis (tacro + MMF + siro triplet regimen) after non-myeloablative/RIC-alloHCT (120). Although these studies provided encouraging results, clinical data and experience with siro are still considered insufficient to recommend its routine use as part of the prophylactic regimen (117). Moreover, a warning has been issued with the use of siro after high dose busulfan-based conditioning regimens due to the increased risk of sinusoidal obstruction syndrome (121).

For almost two decades, *in vivo* T-cell depletion using serotherapies with rabbit anti-T-cell globulin (ATG, ATG-Thymoglobulin^®^ or ATG-Grafalon^®^) (122–126) or alemtuzumab (ALEM, an anti-CD52 monoclonal IgG1 antibody) (127) has also been used to prevent GVHD. Both of these antibody preparations have a long half-life in the human plasma and therefore, once administered as part of the conditioning regimen, they exert their biological effects for several weeks after the graft infusion and induce profound depletion of both host and donor immune cells (128, 129). Moreover, besides the pan T-cell depletion (**Figure 1**), ATG and ALEM also mediated a variety of other immune effects [detailed in other informative reviews (130, 131)]. Several large randomized phase 3 trials have demonstrated the benefit on both a- and cGVHD incidence of adding ATG to standard prophylaxis in the setting of MAC-alloHCT with PBSC (122–126). In a related approach, *ex-vivo* immune cell depletion of the graft (*e.g.* by immunomagnetic positive selection of CD34^+^ stem cells or ALEM in the bag) was also evaluated and proved to be effective to prevent GVHD (132–134). However, a major concern with such an approach is its negative impact on GVL effects and immune recovery.

In recent years, there has been an exponential increase in the number of haplo-alloHCT performed worldwide. This was made possible thanks to the development of innovative platforms for GVHD prevention in this peculiar high alloreactivity setting. Among them, the advent of post-transplant cyclophosphamide (PTCy) has revolutionized this procedure and can be considered as one of the major advances in the field of alloHCT over the past two decades (135–139). This approach, designed by the John Hopkins University group in Baltimore, consists in the administration of (one or) two boluses of high dose cyclophosphamide (Cy, a nitrogen mustard alkylating agent) shortly after alloHCT (day +3 and/or +4) followed by MMF/tacro prophylaxis (starting from day +5). The initial rationale of this strategy mostly assumed to be a cytotoxic and selective depletion of highly proliferative T_eff_ (supposed to be the newly primed alloreactive T cell clones during the first days after the graft infusion) (**Figure 1**), while preserving resting hematopoietic stem cells and non-alloreactive T cells (such as anti-infectious memory T cells) (135). Additional researches further demonstrated that PTCy also induces central tolerance by additional intrathymic clonal deletion of alloreactive T cell precursors (140, 141). Moreover, it was recently suggested that beyond these effects on T_eff_, PTCy-mediated protection against GVHD also (and mainly) relies on the promotion of T_reg_ and the induction of tolerance (135, 141). T_reg_ are indeed less sensitive than T_eff_ to the Cy cytotoxic effects due to their higher expression of aldehyde dehydrogenase (the major detoxifying enzyme for cyclophosphamide) (142). In murine PTCy haplo-alloHCT models, Kanakry et al. showed that PTCy does not completely eliminate alloreactive T_eff_, but instead alters T-cell response to alloantigens and induces the rapid and preferential recovery and expansion of T_reg_ (142). Evidence for the pivotal role of T_reg_ in PTCy-mediated immune tolerance is also illustrated by the development of severe and fatal GVHD when FoxP3+ T_reg_ are depleted (143). Going back to clinical studies, the pioneer pilot trial with the PTCy strategy led by the Baltimore group reported a very low incidence of grade III–IV aGVHD (10%) in patients transplanted with HLA-haploidentical BM after non-myeloablative conditioning regimen (138). Similar encouraging results were further observed by numerous other groups, even using PBSC as the stem cell source and more intensive conditioning regimens (139, 144). Beyond haplo-alloHCT, PTCy recently starts gaining popularity in other settings, including HLA-matched sibling/unrelated donor and HLA-mismatched unrelated donor alloHCT (145). Recently, in a large multicenter phase III trial comparing several novel immunosuppressive prophylactic regimens (PTCy + tacro +MMF; tacro + MMF + bortezomib; tacro + MMF + maraviroc) with the contemporary standard tacro + MTX scheme after RIC-alloHCT, PTCy + tacro + MMF appeared to be the most promising intervention, yielding the best GvHD-free, relapse-free survival (GRFS) (146). It is currently unknown whether another combination (*i.e.* MMF/siro) can be as effective as MMF/CNI in addition to PTCy in haplo-alloHCT, or even if PTCy can be safely used as a single agent after HLA-identical sibling transplantation. It is the subject of numerous investigations.

### Developing Strategies for aGVHD Prevention 

The deeper understanding of aGVHD immunobiology has facilitated the diversification of preventive strategies, and many novel approaches are currently under investigation (6, 29, 116). The concrete clinical goal of aGVHD prophylaxis after alloHCT is to prevent or at least to significantly reduce the damage to target tissues induced by alloreactive immune responses in order to decrease the risk of clinically relevant organ dysfunction leading to “clinical aGVHD”. To achieve this objective, the current strategies being developed/under investigation for limiting aGVHD after alloHCT can be categorized according to three main areas of intervention: (1) limitation of donor-derived immune cell alloreactivity, (2) promotion of immune tolerance, and (3) modulation of the target tissue environment to make it less prone to but rather more resistant to aGVHD immunopathology and to improve regenerative properties. Given the large number of strategies under development, it is difficult to cover them all. Here, we have chosen to present some of those which have already reached clinical trials and which seem to be the most promising in our opinion (**Table 1**).

**Table 1:** Developing strategies for aGVHD prevention.

		T_eff_ depletion	Inhibition of signal 1 of T_eff_ activation	Inhibition of signal 2 of T_eff_ activation	Inhibition of signal 3 of T_eff_ activation	Inhibition of T_eff_ trafficking	Promotion of immune tolerance	Modulation of microenvironment
**Developing strategies**	**Ongoing clinical trial**	**Main putative mechanisms of action**						
*Ex vivo* depletion of TCR*αβ*+/CD19+ donor cells [phase I–II (147)]	NCT04088760 (phase II) NCT02508038 (phase I)	X						
*Ex vivo* depletion of CD45RA^+^ naive T cells [phase II (148)]		X						
*Ex vivo* photodepletion of anti-host reactive donor T cells (Kiadis) [phase II (149)]	NCT02999854 (phase III)	X					X	
Proteasome inhibitors (bortezomib) [phase I–II (146, 150, 151)]	NCT03945591 (phase II) NCT03082677 (phase II) NCT01991301 (phase I) NCT02145403 (phase I–II)		X			X	X	X
*α*-CTLA-4 Ig (abatacept, belatacept) [phase II (152–156)]	NCT02867800 (phase I) NCT01743131 (phase II) NCT04380740 (phase II)			X				
Anti-IL-6 receptor antibody (tocilizumab) [phase I–II (52, 53)]	NCT03434730 (phase II)				X			X
Janus kinases inhibitors (anti-JAK1/2 ruxolitinib, and baricitinib; anti-JAK1 itacitinib)	NCT02806375 (phase I–II) NCT04131738 (phase I) NCT04127721 (phase II) NCT03755414 (phase I) NCT03320642 (phase I)				X		X	X
Demethylating agents (5-azacytidine, decitabine) [phase I–II (157)]	NCT00813124 (phase II) NCT01758367 (phase I–II)	X	X	X	X	X	X	X
Histone deacetylase inhibitors (vorinostat, panobinostat) [phase I–II (158, 159)]	NCT03842696 (phase I–II) NCT03842696 (phase I–II) NCT02588339 (phase II)	X	X	X	X	X	X	X
CCR5 blocker (maraviroc) [phase I–II (74, 146, 160)]	NCT02799888 (phase II)					X		
*α*4*β*7 integrin blocker (vedolizumab) (phase I–II) (161)	NCT03657160 (phase III)					X		
Low dose IL-2 [phase I (162, 163)]	NCT02659657 (phase II)						X	
T_reg_ infusion [phase I–II (reviewed in (164)]	NCT01795573 (phase I) NCT03977103 (phase II) NCT04013685 (phase I)						X	
Mesenchymal stromal cells [phase I–II (reviewed in (165))]	NCT02270307 (phase II–III) NCT01045382 (phase II) NCT04247945 (phase II–III)						X	
iNKT cells [αgalCer, phase II (166) , TLI conditioning (129)]	NCT03605953 NCT00631072						X	
Recombinant urate-oxidase [phase I (167)]	–							X
Alpha-1-antitrypsin	NCT03805789 (phase II-III)							X
Keratinocyte growth factor [phase I-II (168, 169)]	–							X
Probiotics and fecal material transplantation [phase I-III (170–172)]	NCT03720392 (phase II)							X
Prebiotics	NCT02805075 (phase I) NCT02763033 (phase II)							X

Strategies Aimed at Limiting Alloreactivity of Donor Immune Cells (Mainly T Cells) Against Host Tissues (red); Strategies Aimed at Promoting Immune Tolerance (blue); Strategies Aimed at Modulating Target Tissue Environment (green).

#### Strategies Aimed at Limiting Alloreactivity of Donor Immune Cells (Mainly T Cells) Against Host Tissues 

As donor T_eff_ are main causative agents of aGVHD, huge efforts have been made to optimize and refine donor T_eff_ depleting approaches, *e.g.* by selectively depleting specific T-cell subpopulations. In particular, the selective depletion of TCR*αβ*+ cells, of naive T cells, or even of activated alloreactive T cells (*e.g.* with *ex vivo* photodepletion of anti-host reactive donor T cells) has demonstrated encouraging results for aGVHD prevention (147–149). Besides T_eff_ depleting approaches, strategies aimed at functionally interfering with T_eff_ activation (signal 1, 2 and/or 3, **Figure 1**), intracellular signaling pathways, metabolism and homing properties are also developing as well as gene editing approaches.

As described above, signal transduction downstream of TCR activation (signal 1) in T_eff_ occurs through multiple pathways that result in the nuclear translocation of key transcription factors, including NFAT, NF-*κ*B and AP1. Blockade of the NFAT calcium-dependent transduction pathway with CNI (CSA or tacro) is universally used as standard GVHD prophylaxis. Inhibition of the NF-*κ*B pathway also recently appeared as an interesting approach. Proteasome inhibitors, such as bortezomib (BOR), have been shown to suppress NF-*κ*B activation (in part by reducing the degradation of its inhibitory protein I*κ*B*α*) and were reported to confer protection against GVHD in mouse models (39). Moreover, by reducing the degradation of many other intracellular proteins, blocking the proteasome also has an impact on T-cell chemotaxis, secretion of inflammatory cytokines, APC functions and promote T_reg_ (173). Based on these observations, the early addition of short-course BOR (on days +1, +4, and +7 after alloHCT) to standard tacro/MTX has been assessed in phase I–II clinical trials and provided encouraging results (150). However, in a large open-label three-arm phase 2 randomized trial comparing conventional Tacro/MTX *vs.* BOR/Tacro/MTX and *vs.* BOR/Tacro/Siro after UD RIC-alloHCT, BOR-based regimens failed to show an improvement in day +180 aGVHD incidence (32.6, 31.1 and 21%, respectively) (151). Similarly, in another large prospective phase II study comparing several novel prophylactic regimens with contemporary MTX/tacro controls, the addition of BOR to standard MTX/tacro in RIC-alloHCT did not result in lower aGVHD incidence (146). Combination of BOR with other agents, such as PTCy, as well as use of other proteasome inhibitors (carfilzomib, ixazomib) is currently under investigation (**Table 1**).

Targeting costimulatory signals at the APC/T-cell interface (signal 2) has also been investigated as aGVHD prophylaxis for several years. Of all these strategies, the one that has reached the more advanced stage of development concerns CTLA4-Ig (abatacept, belatacept). Addition of abatacept to background CNI-based aGVHD prophylaxis in the setting of alloHCT with HLA-matched donor has produced promising results in phase I–II clinical trials (152, 153). Addition of abatacept to the PTCy platform is also under investigation in the setting of haplo-alloHCT for non-malignant disorders (154, 155). Moreover, unlike T-cell anergy, recent data have shown that NK cell cytotoxicity is not altered, but even enhanced in the presence of CTLA4-Ig. This makes the CTLA4-Ig approach an interesting strategy for reinforcing GVT effects while still limiting aGVHD risks in the setting of HLA-mismatched donor (haplo) transplantation. This hypothesis prompted several groups to study the CTLA4Ig sequential primed donor lymphocyte (DLI) infusion protocols after PTCy-based haplo-alloHCT as adoptive immunotherapy in patients with advanced malignant disorders (156). Of note, one issue with targeting CD80/CD86 with CTLA4-Ig may be associated with concurrent undesired blockade of tolerogenic CTLA4-dependent signaling to T_reg_ and APCs. Hence, CD28-specific inhibition is under investigation in preclinical studies (44).

Different strategies that target signal 3 of T-cell activation by blocking cytokines or their receptors were also tested in clinical studies (42, 47). Among them, blockade of IL-2 signaling with monoclonal antibodies binding to the IL-2 receptor α-chain CD25 (*e.g*. basiliximab, daclizumab, inolimomab) was unfortunately discouraged for controlling aGVHD since it was reported to be associated with increased GVHD-related mortality (174, 175). This is likely due to the negative impact of IL-2 blockade on suppressive T_reg_ since IL-2 is not only crucial for T_eff_ expansion but also for T_reg_ homeostasis.

IL-6, TNF-*α*, and IL-1*β* are important pro-inflammatory cytokines in aGVHD pathogenesis. Addition of Tocilizumab (an anti-IL-6 receptor monoclonal antibody) to CNI/MTX prophylaxis has been tested in phase I–II studies and has been shown to be associated with a very low incidence of grades II–IV aGVHD (<15%) (52, 53). However, these promising results have to be confirmed in larger phase III studies. By contrast, inhibition of TNF-*α* or IL-1*β* added to standard GVHD prophylaxis failed to prevent aGVHD (176, 177). Several additional cytokines (such as IL-12, IL-23, GM-CSF, *etc.*) have also been implicated in aGVHD pathogenesis, and their inhibition should also be evaluated in the future.

T cells respond to many inflammatory cytokines (including IL-6) through JAK/STAT pathways. As described above, several studies have shown that the inhibition of JAK1/2 pathways (*i.e.* with ruxolitinib and baricitinib, two JAK1/2 inhibitors, or with itacitinib, a selective JAK1 inhibitor) prevented aGVHD in preclinical model (50) and was efficacious for controlling steroid refractory-aGVHD in patients (26). Further ongoing studies are investigating the use of this molecule and other JAK1/JAK2 inhibitors for aGVHD prevention (**Table 1**). Itacitinib, which inhibits JAK1 while sparing JAK2, is expected to have reduced myelosuppressive activity compared to broader specificity JAK inhibitors.

Encouraging results also come from epigenetic modifiers [*e.g.* demethylating agents such as 5-azacytidine, decitabine, histone deacetylase inhibitors (HDACi)] which can exert pleiotropic effects on aGVHD reactions, not only on the fate of T_eff_ but also on other immune cells (such as T_reg_ and DCs) (157–159). For example, the addition of vorinostat (a HDACi) to standard GVHD prophylaxis after alloHCT with HLA-matched donors was examined in two phase 2 clinical trials (158, 159). Both studies showed that vorinostat was well tolerated and was associated with a low incidence of aGVHD (grade II-IV aGVHD less than 25%, and grades III–IV less than 10%). Additional advantages of such approaches lie in the fact that, besides their immunomodulatory effects, these molecules (demethylating agents and HDACi) can also exert anti-tumor activity, therefore offering opportunities for mitigating GVHD while enhancing anti-tumor effects.

Interfering with the homing of T_eff_ towards target organs can be viewed as an additional strategy for preventing aGVHD. A phase I–II study indeed investigated the addition of maraviroc (a CCR5 antagonist) to standard tacro/MTX after RIC-alloHCT in adults and demonstrated a low incidence of visceral (GI and liver) grades II–IV aGVHD (14.7%) (74). Similar encouraging results were observed in pediatric patients (160). Nevertheless, a recent multicenter phase II trial comparing several new prophylactic regimens with contemporary MTX/tacro controls in RIC-alloHCT showed that, when added to standard MTX/tacro, maraviroc did not result in lower GVHD rates compared to PTCy or BOR (146). The redundant mechanisms in the signaling of chemokines/chemokine receptors may be an explanation for the limited effectiveness of strategies based on blocking just a single chemokine receptor. Integrins also represent attractive potential targets for novel preventive therapies against GVHD. Low incidences of grades II–IV overall and lower-intestinal aGVHD (19 and 14% at day 100, respectively) were recently observed in a phase Ib study in which patients received vedolizumab (an antibody directed againts *α*4*β*7 integrin) in combination with standard tacro/MTX (161). A large phase III randomized placebo control trial evaluating vedolizumab added to standard aGvHD prophylaxis is currently recruiting (NCT03657160, **Table 1**).

Finally, since T cells consume a lot of energy during aGVHD, it can also be envisaged that targeting metabolic pathways and subverting the use of T-cell energy could offer other potential innovative preventive strategies to explore in the future. The challenge will be to make these molecules specific enough to avoid important toxicities.

#### Strategies Aimed at Promoting Immune Tolerance

Rather than trying to decrease the reactivity of the donor immune cells, another way of preventing aGVHD after alloHCT may be through the promotion of tolerance between the donor immune cells and the recipient, by strengthening the tolerogenic arm of the immune system. Indeed, cell-based approaches to promote immune tolerance have shown encouraging results. In our view, the most promising are T_reg_, iNKT, and MSC-based therapies.

Early clinical trials with iT_reg_ infusion in patients have shown promising results for aGVHD prevention (178–180), [reviewed in (164)]. Nevertheless, the major problem with the clinical transfer of T_reg_ is the difficulty of reaching a sufficient number of T_reg_ with good purity to infuse and of ensuring that the transferred cells persist and retain their tolerogenic properties in the inflammatory context of aGVHD (181). Strategies aimed at promoting T_reg_ proliferation in the donor before T_reg_ donation, for example by pretreating the donor with TNF superfamily receptors DR3 agonists, have been reported to be effective in murine models (182) but have not yet been explored in humans. The scientific community is currently focusing on examining approaches to promote *in vivo* T_reg_ expansion and stability within the recipient. In particular, the high sensitivity of T_reg_ to IL-2 (determined by their constitutive expression of CD25) makes treatment with low doses of this cytokine an interesting approach. A phase I–II study investigated the administration of ultra-low dose IL-2 (100,000–200,000 IU/m^2^, 3 times/week) after alloHCT and reported promising results in terms of safety and low incidence of aGVHD (0/16 patients experienced grade II–IV aGVHD) (162). Another study using a similar approach is underway in China (NCT02659657, **Table 1**). However, in another trial administration of low doses of IL-2 in addition to tacro/siro for GVHD prophylaxis failed to prevent aGVHD despite resulting in higher T_reg_ levels (163).

As with iT_reg_, adoptive transfer of IL-10/TGF-*β* producing Tr1 cells is gradually being seen as a new option for the prevention of aGVHD. A pilot phase 1 clinical trial evaluating the safety of Tr1 cell co-transplantation in pediatric patients in an HLA-mismatched donors setting is currently being planned (NCT03198234).

A high content of iNKT cells in the transplant has been reported to be associated with a reduced risk of aGVHD in clinical studies (183). Thus, protocols for promoting the expansion of iNKT cells (*e.g.* through *ex* or *in vivo* manipulations) appear as attractive novel strategies to explore in order to prevent aGVHD. Clinical studies involving the *ex vivo* expansion of iNKT cell populations are underway (NCT00631072, NCT03605953, **Table 1**). Recently, it was reported that RGI-2001, a CD1-binding synthetic derivative of alpha-galactosylceramide, activates and expands iNKT cells *in vivo* (166). Conditioning regimens that foster the induction of iNKT cells, such as total lymphoid irradiation, are also being considered (129).

MSCs are multipotent progenitor cells that reside within the BM microenvironnement and several other connective tissues such as the adipose tissue, the umbilical cord, and placenta membranes. Among a wide variety of functions, MSCs also have a multiplicity of immunomodulatory and anti-inflammatory properties, making them attractive candidates to consider as cell-based therapies to prevent aGVHD. Moreover, MSCs are hypoimmunogenic and can therefore be derived from third-party HLA-mismatched donors. A number of preclinical studies using various animal models have evaluated the effectiveness of MSCs in alleviating GVHD. However, results were mixed, with some studies reporting benefits (184), while others did not (185). Several factors, including MSC tissue of origin (BM, adipose tissue, cord blood, placental membranes), cell dose, timing of infusion and pre-activated MSC status likely influenced the results and caused heterogeneity between studies. Pilot clinical studies have also suggested a potential role for MSCs in preventing GVHD (186–188), [reviewed in (165)]. Further studies are currently underway to more precisely assess the impact of MSC co-transplantation on aGVHD (**Table 1**).

#### Strategies Aimed at Modulating Target Tissue Environment 

Beyond targeting T_eff_ and promoting immune tolerance, approaches aimed at controlling target tissue environment to make it less pro-inflammatory and/or aimed at strengthening its mechanisms of resilience, repair and regeneration (“tissue tolerance”) may be considered as complementary strategies to be exploited to mitigate aGVHD clinical severity.

Among others, molecules aimed at reducing danger signal production (*e.g.* recombinant urate-oxidase, alpha-1-antitrypsin) are currently under investigation (167, 189, 190).

Tissue-protective/regenerative approaches that promote the healing of aGVHD-related tissue damages have also emerged as promising complementary strategies to standard aGVHD immuno-prophylaxis. As mentioned above, KGF, R-Spo1, IL-22, and GLP-2 were reported to be protective during GI aGVHD (97–101). To the best of our knowledge, among all these molecules, only KGF has been tested to date in clinical trials for aGVHD prevention. Two phase 1/2 randomized, double-blind, placebo-controlled studies tested peri-transplant palifermin (KGF) administration in combination with standard prophylaxis (168, 169). Both of them failed to demonstrate benefit in terms of reduction of severe grades III–IV aGVHD. Clinical trials on IL-22 IgG2-Fc (NCT02406651) and GLP-2 (Teduglutide, NCT04290429) for the treatment of GI aGVHD are underway, and it is plausible that these drugs will soon be tested for aGVHD prophylaxis. 

The accumulation of evidence on the involvement of the commensal microbiota in intestinal tissue homeostasis and immune tolerance post-alloHCT has also recently opened up the concept of manipulating the gut microbiota as an innovative approach to prevent aGVHD. Several strategies under study include careful risk-balanced use of broad-spectrum antibiotics, dietary or pharmaceutical interventions to limit growth of noxious bacterial taxa [*i.e.* eviction of lactose (191) or enteral immunoglobulin administration (192)] and direct transfer of living microbial species using fecal material transplantation (FMT) (170, 171) or selective transfer of microbial consortia (probiotics) (172, 193, 194). Some of them have already reached clinical trials (see **Table 1**). As such, FMT appears to be a promising approach to improve microbiota diversity in alloHCT patients and to limit aGVHD (170, 171). However, considering the highly immunocompromised status of alloHCT patients, safety of FMT should be carefully established in this specific population, particularly regarding risk of bacterial translocation, septicemia and norovirus infection (195, 196). The modulation of the microbiome–metabolome axis with prebiotic/postbiotic interventions is also under investigation. Among others, the microbiota-derived SCFA butyrate appears as an important metabolite for intestinal homeostasis and immune tolerance after alloHCT. Interestingly, one approach for stimulating microbial SCFA production could be *via* dietary supplementation with non-digestible carbohydrates that can be metabolized by selected commensal gut bacteria. Such strategy is currently being explored in clinical trials in alloHCT patients (**Table 1**). Among them, a phase II clinical trial is testing the safety and early efficacy for GVHD prevention of an oral dietary supplement containing potato-based starch [which was reported to increase microbial butyrate production in healthy volunteers (197)] (NTC02763033). Besides SCFAs, roles of other microbial metabolites (such as indole derivatives, peptides derived from bile acids, aryl hydrocarbon receptor ligands, polyamine, plasmalogens) would also be interesting to explore in the future.

## Conclusion and Perspective

AGVHD is a severe complication after allogeneic stem cell transplantation. It results from a highly deregulated immune process, involving a complex network of multiple molecular and cellular mediators and effectors causing end-organ damages mainly to the skin, GI tract and/or liver. Despite prophylactic measures, aGVHD still develops in about 20–50% of transplanted patients, making it an unmet medical need in alloHCT survivorship research. Improved understanding of the pathology of aGVHD has led to the development of novel strategies to optimize its prevention, with some of them appearing particularly promising based on early data from clinical trials. However, these and other new strategies that will be developed in the future will have to be tested in prospective phase 3 trials before they can become standard. Standardization of aGVHD definition criteria and severity grading system using the validated MAGIC criteria will be vitally important to guarantee the quality, reproducibility and interpretation of these future clinical studies.

Theoretically, it would be logical to think that the combination of multiple approaches targeting several aGVHD immunopathological pathways would ultimately provide a complete suppression of aGVHD. However, the complete abrogation of donor-derived immunity after alloHCT is clinically irrelevant, as this would seriously compromise the engraftment, anti-infectious immune reconstitution as well as the beneficial GVT effects. The ideal step in the future would rather be to provide a personalized risk-stratified aGVHD prophylaxis regimen for each patient, reserving intensive immunosuppressive regimens for patients at high risk for aGVHD and avoiding excessive immunosuppression for those at a low risk for aGVHD. To make this approach feasible, the development of future algorithms to improve the accuracy of aGVHD risk prediction will be an essential prerequisite. Algorithms may be based on HLA disparities and other factors, including predictive biomarkers, clinical predictive factors and genetic variants associated with increased risk of aGVHD. Recipient and/or donor single nucleotide polymorphisms (SNPs) for chemokines, cytokines, costimulatory molecules, and micro-RNAs (miRNAs) would also likely allow transplant physicians to identify specific immune profiles predictors of aGVHD in the future. However, these analyses are not yet accessible for a routine assessment in daily clinical practice.

Unlike immunosuppressive strategies, approaches aimed at modulating the interactions between the host and gut microbiota and/or promoting the regenerative properties of the target tissue of aGVHD would likely not increase the risk of non-engraftment or relapse after alloHCT and would therefore appear to be interesting complementary approaches to combine with classical GVHD immunosuppressive prophylaxis. At present, little is known about the precise mechanisms of host–microbiota cross-talk and about tissue-specific tolerance to diseases, but it is a topic of growing interest and intense research.

## Author Contributions

BV and LC are co-first authors. TK and SS are co-last authors. All authors contributed to the article and approved the submitted version.

## Funding

The review was supported by funds from the FBC (ref 2017-037), the FNRS (ref 7.4607.19), King Baudouin Foundation (ref J1813410), the Anti-Cancer Center and the Leon Fredericq Foundation from the University of Liege. **Table 1** and **Figure 1** were adapted and republished from the Belgian Journal of Hematology (Belg J Hematol 2020;11(4):159–173) (35).

## Conflict of Interest

HS has received travel grants and/or speaker honoraria from Incyte, Janssen, Jazz Pharmaceuticals, Novartis, Takeda, Celgene and Abbvie. She also received research funding from Novartis for an investigator-initiated study. FB has received travel grants and/or speaker honoraria from Celgene, AbbVie, Novartis, Pfizer and Sanofi. CG has received travel grants and/or speaker honoraria from Amgen, Incyte, Janssen, Astellas, Novartis, Celgene. TK has received travel grants and/or speaker honoraria (never on personal account) from Celgene, BMS, Roche, Merck, Novartis, Pfizer and Sanofi. SS has received travel grants and/or speaker honoraria from Novartis, Fresenuis-Kabi, Gilead, Sanofi, Jazz Pharmaceuticals, Merck, BMS and Celgene. She also received a grant from Gilead for a basic research project.

The remaining authors declare that the research was conducted in the absence of any commercial or financial relationships that could be construed as a potential conflict of interest.

## References

[1] Duarte RF Labopin M Bader P Basak GW Bonini C Chabannon C Indications for haematopoietic stem cell transplantation for haematological diseases, solid tumours and immune disorders: current practice in Europe. Bone Marrow Transplant (2019) 54(10):1525–52. 10.1038/s41409-019-0516-2 30953028

[2] Passweg JR Baldomero H Chabannon C Basak GW Corbacioglu S Duarte R The EBMT activity survey on hematopoietic-cell transplantation and cellular therapy 2018: CAR-T’s come into focus. Bone Marrow Transplant (2020) 55(8):1604–13. 10.1038/s41409-020-0826-4 PMC7391287 32066864

[3] Filipovich AH Weisdorf D Pavletic S Socie G Wingard JR Lee SJ National Institutes of Health Consensus Development Project on Criteria for Clinical Trials in Chronic Graft-versus-Host Disease: I. Diagnosis and Staging Working Group Report. Biol Blood Marrow Transplant (2005) 11(12):945–56. 10.1016/j.bbmt.2005.09.004 16338616

[4] Jagasia MH Greinix HT Arora M Williams KM Wolff D Cowen EW National Institutes of Health Consensus Development Project on Criteria for Clinical Trials in Chronic Graft-versus-Host Disease: I. The 2014 Diagnosis and Staging Working Group Report. Biol Blood Marrow Transplant (2015) 21(3):389–401. 10.1016/j.bbmt.2015.02.025 25529383 PMC4329079

[5] Schoemans HM Lee SJ Ferrara JL Wolff D Levine JE Schultz KR EBMT–NIH–CIBMTR Task Force position statement on standardized terminology & guidance for graft-versus-host disease assessment. Bone Marrow Transplant (2018) 53(11):1401–15. 10.1038/s41409-018-0204-7 PMC6786777 29872128

[6] Zeiser R Blazar BR Acute Graft-versus-Host Disease — Biologic Process, Prevention, and Therapy. Longo DL, editor. N Engl J Med (2017) 377(22):2167–79. 10.1056/NEJMra1609337 PMC6034180 29171820

[7] Jagasia M Arora M Flowers MED Chao NJ McCarthy PL Cutler CS Risk factors for acute GVHD and survival after hematopoietic cell transplantation. Blood (2012) 119(1):296–307. 10.1182/blood-2011-06-364265 22010102 PMC3251233

[8] Harris AC Young R Devine S Hogan WJ Ayuk F Bunworasate U International, Multicenter Standardization of Acute Graft-versus-Host Disease Clinical Data Collection: A Report from the Mount Sinai Acute GVHD International Consortium. Biol Blood Marrow Transplant (2016) 22(1):4–10. 10.1016/j.bbmt.2015.09.001 26386318 PMC4706482

[9] Glucksberg H Storb R Fefer A Buckner CD Neiman PE Clift RA Clinical manifestations of graft-versus-host disease in human recipients of marrow from hl-a-matched sibling donor,s. Transplantation (1974) 18(4):295–304. 10.1097/00007890-197410000-00001 4153799

[10] Przepiorka D Weisdorf D Martin P Klingemann HG Beatty P Hows J 1994 Consensus Conference on Acute GVHD Grading. Bone Marrow Transplant (1995) 15(6):825–8. 7581076

[11] Rowlings PA Przepiorka D Klein JP Gale RP Passweg JR Jean Henslee-Downey P IBMTR Severity index for grading acute graft-versus-host disease: retrospective comparison with glucksberg grade. Br J Haematol (1997) 97(4):855–64. 10.1046/j.1365-2141.1997.1112925.x 9217189

[12] Schoemans HM Goris K Van Durm R Fieuws S De Geest S Pavletic SZ The eGVHD App has the potential to improve the accuracy of graft-versus-host disease assessment: a multicenter randomized controlled trial. Haematologica (2018) 103(10):1698–707. 10.3324/haematol.2018.190777 PMC6165809 29903762

[13] Schoemans HM Goris K Van Durm R Vanbrabant K De Geest S Maertens J Accuracy and usability of the eGVHD app in assessing the severity of graft-versus-host disease at the 2017 EBMT annual congress. Bone Marrow Transplant (2018) 53(4):490–4. 10.1038/s41409-017-0017-0 29330389

[14] Clave E Busson M Douay C Peffault de Latour R Berrou J Rabian C Acute graft-versus-host disease transiently impairs thymic output in young patients after allogeneic hematopoietic stem cell transplantation. Blood (2009) 113(25):6477–84. 10.1182/blood-2008-09-176594 19258596

[15] Castermans E Hannon M Dutrieux J Humblet-Baron S Seidel L Cheynier R Thymic recovery after allogeneic hematopoietic cell transplantation with non-myeloablative conditioning is limited to patients younger than 60 years of age. Haematologica (2011) 96(2):298–306. 10.3324/haematol.2010.029702 20934996 PMC3031699

[16] Pagliuca S Michonneau D Sicre de Fontbrune F Sutra del Galy A Xhaard A Robin M Allogeneic reactivity–mediated endothelial cell complications after HSCT: a plea for consensual definitions. Blood Adv (2019) 3(15):2424–35. 10.1182/bloodadvances.2019000143 PMC6693000 31409584

[17] Mathew NR Vinnakota JM Apostolova P Erny D Hamarsheh S Andrieux G Graft-versus-host disease of the CNS is mediated by TNF upregulation in microglia. J Clin Invest (2020) 130(3):1315–29. 10.1172/JCI130272 PMC7269577 31846439

[18] Belle L Zhou V Stuhr KL Beatka M Siebers EM Knight JM Host interleukin 6 production regulates inflammation but not tryptophan metabolism in the brain during murine GVHD. JCI Insight (2017) 2(14):e93726. 10.1172/jci.insight.93726 PMC5518565 28724796

[19] Ruggiu M Cuccuini W Mokhtari K Meignin V Peffault de Latour R Robin M Case report: Central nervous system involvement of human graft versus host disease: Report of 7 cases and a review of literature. Medicine (2017) 96(42):e8303. 10.1097/MD.0000000000008303 29049232 PMC5662398

[20] Flowers MED Inamoto Y Carpenter PA Lee SJ Kiem H-P Petersdorf EW Comparative analysis of risk factors for acute graft-versus-host disease and for chronic graft-versus-host disease according to National Institutes of Health consensus criteria. Blood (2011) 117(11):3214–9. 10.1182/blood-2010-08-302109 PMC3062319 21263156

[21] Loiseau P Busson M Balere M-L Dormoy A Bignon J-D Gagne K HLA Association with Hematopoietic Stem Cell Transplantation Outcome: The Number of Mismatches at HLA-A, -B, -C, -DRB1, or -DQB1 Is Strongly Associated with Overall Survival. Biol Blood Marrow Transplant (2007) 13(8):965–74. 10.1016/j.bbmt.2007.04.010 17640601

[22] Saliba RM Couriel DR Giralt S Rondon G Okoroji G-J Rashid A Prognostic value of response after upfront therapy for acute GVHD. Bone Marrow Transplant (2012) 47(1):125–31. 10.1038/bmt.2011.41 PMC4114039 21383686

[23] Westin JR Saliba RM De Lima M Alousi A Hosing C Qazilbash MH Steroid-Refractory Acute GVHD: Predictors and Outcomes. Adv Hematol (2011) 2011:601953. 10.1155/2011/601953 22110505 PMC3216266

[24] Inamoto Y Martin PJ Storer BE Mielcarek M Storb RF Carpenter PA Response endpoints and failure-free survival after initial treatment for acute graft-versus-host disease. Haematologica (2014) 99(2):385–91. 10.3324/haematol.2013.093062 PMC3912972 24056814

[25] Socié G Vigouroux S Yakoub-Agha I Bay J-O Fürst S Bilger K A phase 3 randomized trial comparing inolimomab vs usual care in steroid-resistant acute GVHD. Blood (2017) 129(5):643–9. 10.1182/blood-2016-09-738625 27899357

[26] Zeiser R von Bubnoff N Butler J Mohty M Niederwieser D Or R Ruxolitinib for Glucocorticoid-Refractory Acute Graft-versus-Host Disease. N Engl J Med (2020) 382(19):1800–10. 10.1056/NEJMoa1917635 32320566

[27] Styczyński J Tridello G Koster L Iacobelli S van Biezen A van der Werf S Death after hematopoietic stem cell transplantation: changes over calendar year time, infections and associated factors. Bone Marrow Transplant (2020) 55(1):126–36. 10.1038/s41409-019-0624-z PMC6957465 31455899

[28] Zeiser R Socié G Blazar BR Pathogenesis of acute graft-versus-host disease: from intestinal microbiota alterations to donor T cell activation. Br J Haematol (2016) 175(2):191–207. 10.1111/bjh.14295 27619472

[29] Zeiser R Biology-driven developments in the therapy of acute graft-versus-host disease. Hematology (2018) 18(1):236–41. 10.1182/asheducation-2018.1.236 PMC6245989 30504316

[30] Ferrara JLM Chaudhry MS GVHD: biology matters. Blood Adv (2018) 2(22):3411–7. 10.1182/bloodadvances.2018020214 PMC6258915 30482771

[31] Aversa F Tabilio A Velardi A Cunningham I Terenzi A Falzetti F Treatment of high-risk acute leukemia with T-cell-depleted stem cells from related donors with one fully mismatched HLA haplotype. N Engl J Med (1998) 339(17):1186–93. 10.1056/NEJM199810223391702 9780338

[32] DeWolf S Sykes M Alloimmune T cells in transplantation. J Clin Invest (2017) 127(7):2473–81. 10.1172/JCI90595 PMC5490749 28628037

[33] Wang Y Singh NK Spear TT Hellman LM Piepenbrink KH McMahan RH How an alloreactive T-cell receptor achieves peptide and MHC specificity. Proc Natl Acad Sci U S A (2017) 114(24):E4792–801. 10.1073/pnas.1700459114 PMC5474831 28572406

[34] Martin PJ Levine DM Storer BE Warren EH Zheng X Nelson SC Genome-wide minor histocompatibility matching as related to the risk of graft-versus-host disease. Blood (2017) 129(6):791–8. 10.1182/blood-2016-09-737700 PMC5301826 27872059

[35] Vandenhove B Canti L Schoemans H Beguin Y Baron F Willems E Acute graft-versus-host disease: diagnosis, pathophysiology and prevention. Belg J Hematol (2020) 11(4):159–73.

[36] Bhattacharyya ND Feng CG Regulation of T Helper Cell Fate by TCR Signal Strength. Front Immunol (2020) 11:624. 10.3389/fimmu.2020.00624 32508803 PMC7248325

[37] Storb R Deeg HJ Whitehead J Appelbaum F Beatty P Bensinger W Methotrexate and Cyclosporine Compared with Cyclosporine Alone for Prophylaxis of Acute Graft versus Host Disease after Marrow Transplantation for Leukemia. N Engl J Med (1986) 314(12):729–35. 10.1056/NEJM198603203141201 3513012

[38] Blanco B Pérez-Simón JA Sánchez-Abarca LI Carvajal-Vergara X Mateos J Vidriales B Bortezomib induces selective depletion of alloreactive T lymphocytes and decreases the production of Th1 cytokines. Blood (2006) 107(9):3575–83. 10.1182/blood-2005-05-2118 16282346

[39] Pai C-CS Hsiao H-H Sun K Chen M Hagino T Tellez J Therapeutic benefit of bortezomib on acute graft-versus-host disease is tissue specific and is associated with interleukin-6 levels. Biol Blood Marrow Transplant (2014) 20(12):1899–904. 10.1016/j.bbmt.2014.07.022 PMC4254314 25064746

[40] Vodanovic-Jankovic S Hari P Jacobs P Komorowski R Drobyski WR NF-kappaB as a target for the prevention of graft-versus-host disease: comparative efficacy of bortezomib and PS-1145. Blood (2006) 107(2):827–34. 10.1182/blood-2005-05-1820 PMC1895627 16174760

[41] Kumar S Leigh ND Cao X The Role of Co-stimulatory/Co-inhibitory Signals in Graft-vs.-Host Disease. Front Immunol (2018) 9:3003. 10.3389/fimmu.2018.03003 30627129 PMC6309815

[42] Hill GR Koyama M Cytokines and Co-stimulation in Acute Graft-versus-Host Disease. Blood (2020) 136(4):418–28. 10.11182/blood.2019000952 PMC7378458 32526028

[43] Wallace PM Johnson JS MacMaster JF Kennedy KA Gladstone P Linsley PS CTLA4Ig treatment ameliorates the lethality of murine graft-versus-host disease across major histocompatibility complex barriers. Transplantation (1994) 58(5):602–10. 10.1097/00007890-199409150-00013 8091487

[44] Watkins BK Tkachev V Furlan SN Hunt DJ Betz K Yu A CD28 blockade controls T cell activation to prevent graft-versus-host disease in primates. J Clin Invest (2018) 128(9):3991–4007. 10.1172/JCI98793 30102255 PMC6118599

[45] Blazar BR Taylor PA Linsley PS Vallera DA In vivo blockade of CD28/CTLA4: B7/BB1 interaction with CTLA4-Ig reduces lethal murine graft-versus-host disease across the major histocompatibility complex barrier in mice. Blood (1994) 83(12):3815–25. 10.1182/blood.V83.12.3815.bloodjournal83123815 7515723

[46] Blazar BR Sharpe AH Taylor PA Panoskaltsis-Mortari A Gray GS Korngold R Infusion of anti-B7.1 (CD80) and anti-B7.2 (CD86) monoclonal antibodies inhibits murine graft-versus-host disease lethality in part via direct effects on CD4+ and CD8+ T cells. J Immunol (1996) 157(8):3250–9. 8871619

[47] Kim S Reddy P Targeting Signal 3 Extracellularly and Intracellularly in Graft-Versus-Host Disease. Front Immunol (2020) 11:722. 10.3389/fimmu.2020.00722 32411139 PMC7198807

[48] Myers DR Wheeler B Roose JP mTOR and other effector kinase signals that impact T cell function and activity. Immunol Rev (2019) 291(1):134–53. 10.1111/imr.12796 PMC6697425 31402496

[49] Mannina D Kröger N Janus Kinase Inhibition for Graft-Versus-Host Disease: Current Status and Future Prospects. Drugs (2019) 79(14):1499–509. 10.1007/s40265-019-01174-1 31359326

[50] Elli EM Baratè C Mendicino F Palandri F Palumbo GA Mechanisms Underlying the Anti-inflammatory and Immunosuppressive Activity of Ruxolitinib. Front Oncol (2019) 9:1186. 10.3389/fonc.2019.01186 31788449 PMC6854013

[51] Delens L Ehx G Somja J Vrancken L Belle L Seidel L In Vitro Th17-Polarized Human CD4+ T Cells Exacerbate Xenogeneic Graft-versus-Host Disease. Biol Blood Marrow Transplant (2019) 25(2):204–15. 10.1016/j.bbmt.2018.10.007 30326279

[52] Drobyski WR Szabo A Zhu F Keever-Taylor C Hebert KM Dunn R Tocilizumab, tacrolimus and methotrexate for the prevention of acute graft-versus-host disease: low incidence of lower gastrointestinal tract disease. Haematologica (2018) 103(4):717–27. 10.3324/haematol.2017.183434 PMC5865423 29351985

[53] Kennedy GA Varelias A Vuckovic S Le Texier L Gartlan KH Zhang P Addition of interleukin-6 inhibition with tocilizumab to standard graft-versus-host disease prophylaxis after allogeneic stem-cell transplantation: a phase 1/2 trial. Lancet Oncol (2014) 15(13):1451–9. 10.1016/S1470-2045(14)71017-4 25456364

[54] Kang S Tanaka T Narazaki M Kishimoto T Targeting Interleukin-6 Signaling in Clinic. Immunity (2019) 50(4):1007–23. 10.1016/j.immuni.2019.03.026 30995492

[55] Wilkinson AN Chang K Kuns RD Henden AS Minnie SA Ensbey KS IL-6 dysregulation originates in dendritic cells and mediates graft-versus-host disease via classical signaling. Blood (2019) 134(23):2092–106. 10.1182/blood.2019000396 31578204

[56] Chen BJ Morris RE Chao NJ Graft-versus-host disease prevention by rapamycin: cellular mechanisms. Biol Blood Marrow Transplant (2000) 6(5A):529–36. 10.1016/S1083-8791(00)70062-0 11071258

[57] Blazar BR Taylor PA Panoskaltsis-Mortari A Vallera DA Rapamycin inhibits the generation of graft-versus-host disease- and graft-versus-leukemia-causing T cells by interfering with the production of Th1 or Th1 cytotoxic cytokines. J Immunol (1998) 160(11):5355–65. 9605135

[58] Zeiser R Leveson-Gower DB Zambricki EA Kambham N Beilhack A Loh J Differential impact of mammalian target of rapamycin inhibition on CD4+CD25+Foxp3+ regulatory T cells compared with conventional CD4+ T cells. Blood (2008) 111(1):453–62. 10.1182/blood-2007-06-094482 PMC2200823 17967941

[59] Tijaro-Ovalle NM Karantanos T Wang H-T Boussiotis VA Metabolic Targets for Improvement of Allogeneic Hematopoietic Stem Cell Transplantation and Graft-vs.-Host Disease. Front Immunol (2019) 10:295. 10.3389/fimmu.2019.00295 30891031 PMC6411635

[60] Nguyen HD Chatterjee S Haarberg KMK Wu Y Bastian D Heinrichs J Metabolic reprogramming of alloantigen-activated T cells after hematopoietic cell transplantation. J Clin Invest (2016) 126(4):1337–52. 10.1172/JCI82587 PMC4811142 26950421

[61] Koyama M Mukhopadhyay P Schuster IS Henden AS Hülsdünker J Varelias A MHC Class II Antigen Presentation by the Intestinal Epithelium Initiates Graft-versus-Host Disease and Is Influenced by the Microbiota. Immunity (2019) 51(5):885–98.e7. 10.1016/j.immuni.2019.08.011 31542340 PMC6959419

[62] Koyama M Kuns RD Olver SD Raffelt NC Wilson YA Don ALJ Recipient nonhematopoietic antigen-presenting cells are sufficient to induce lethal acute graft-versus-host disease. Nat Med (2011) 18(1):135–42. 10.1038/nm.2597 22127134

[63] Toubai T Mathewson ND Magenau J Reddy P Danger Signals and Graft-versus-host Disease: Current Understanding and Future Perspectives. Front Immunol (2016) 7:539. 10.3389/fimmu.2016.00539 27965667 PMC5126092

[64] Jones JM Wilson R Bealmear PM Mortality and gross pathology of secondary disease in germfree mouse radiation chimeras. Radiat Res (1971) 45(3):577–88. 10.2307/3573066 4396814

[65] van Bekkum DW Roodenburg J Heidt PJ van der Waaij D Mitigation of secondary disease of allogeneic mouse radiation chimeras by modification of the intestinal microflora. J Natl Cancer Inst (1974) 52(2):401–4. 10.1093/jnci/52.2.401 4150164

[66] van Bekkum DW Knaan S Role of bacterial microflora in development of intestinal lesions from graft-versus-host reaction. J Natl Cancer Inst (1977) 58(3):787–90. 10.1093/jnci/58.3.787 14265

[67] Fredricks DN The gut microbiota and graft-versus-host disease. J Clin Invest (2019) 129(5):1808–17. 10.1172/JCI125797 PMC6486325 31042160

[68] Socié G Mary J-Y Lemann M Daneshpouy M Guardiola P Meignin V Prognostic value of apoptotic cells and infiltrating neutrophils in graft-versus-host disease of the gastrointestinal tract in humans: TNF and Fas expression. Blood (2004) 103(1):50–7. 10.1182/blood-2003-03-0909 12881313

[69] Schwab L Goroncy L Palaniyandi S Gautam S Triantafyllopoulou A Mocsai A Neutrophil granulocytes recruited upon translocation of intestinal bacteria enhance graft-versus-host disease via tissue damage. Nat Med (2014) 20(6):648–54. 10.1038/nm.3517 24836575

[70] Kittan NA Hildebrandt GC The Chemokine System: A Possible Therapeutic Target in Acute Graft Versus Host Disease. Curr Top Microbiol Immunol (2010) 341:97–120. 10.1007/82_2010_23 20379809

[71] Zheng H Matte-Martone C Li H Anderson BE Venketesan S Sheng Tan H Effector memory CD4+ T cells mediate graft-versus-leukemia without inducing graft-versus-host disease. Blood (2008) 111(4):2476–84. 10.1182/blood-2007-08-109678 PMC2234071 18045967

[72] Chen BJ Deoliveira D Cui X Le NT Son J Whitesides JF Inability of memory T cells to induce graft-versus-host disease is a result of an abortive alloresponse. Blood (2007) 109(7):3115–23. 10.1182/blood-2006-04-016410 PMC1852216 17148592

[73] Yuan J Ren H Shi Y Liu W Prophylaxis of acute graft-versus-host disease by CCR5 blockade combined with cyclosporine A in a murine model. Inflammation Res (2015) 64(2):137–44. 10.1007/s00011-014-0793-6 25556580

[74] Reshef R Luger SM Hexner EO Loren AW Frey NV Nasta SD Blockade of lymphocyte chemotaxis in visceral graft-versus-host disease. N Engl J Med (2012) 367(2):135–45. 10.1056/NEJMoa1201248 PMC3568501 22784116

[75] Dutt S Ermann J Tseng D Liu YP George TI Fathman CG L-selectin and beta7 integrin on donor CD4 T cells are required for the early migration to host mesenteric lymph nodes and acute colitis of graft-versus-host disease. Blood (2005) 106(12):4009–15. 10.1182/blood-2005-06-2339 PMC1895109 16105972

[76] Petrovic A Alpdogan O Willis LM Eng JM Greenberg AS Kappel BJ LPAM (alpha 4 beta 7 integrin) is an important homing integrin on alloreactive T cells in the development of intestinal graft-versus-host disease. Blood (2004) 103(4):1542–7. 10.1182/blood-2003-03-0957 14563643

[77] Caridade M Graca L Ribeiro RM Mechanisms Underlying CD4+ Treg Immune Regulation in the Adult: From Experiments to Models. Front Immunol (2013) 4:378. 10.3389/fimmu.2013.00378 24302924 PMC3831161

[78] Liston A Gray DHD Homeostatic control of regulatory T cell diversity. Nat Rev Immunol (2014) 14(3):154–65. 10.1038/nri3605 24481337

[79] Schiavon V Duchez S Branchtein M How-Kit A Cassius C Daunay A Microenvironment tailors nTreg structure and function. Proc Natl Acad Sci U S A (2019) 116(13):6298–307. 10.1073/pnas.1812471116 PMC6442590 30846549

[80] Chinen T Kannan AK Levine AG Fan X Klein U Zheng Y An essential role for the IL-2 receptor in T(reg) cell function. Nat Immunol (2016) 17(11):1322–33. 10.1038/ni.3540 PMC5071159 27595233

[81] Cohen JL Trenado A Vasey D Klatzmann D Salomon BL CD4(+)CD25(+) immunoregulatory T Cells: new therapeutics for graft-versus-host disease. J Exp Med (2002) 196(3):401–6. 10.1084/jem.20020090 PMC2193933 12163568

[82] Hannon M Lechanteur C Lucas S Somja J Seidel L Belle L Infusion of clinical-grade enriched regulatory T cells delays experimental xenogeneic graft-versus-host disease. Transfusion (2014) 54(2):353–63. 10.1111/trf.12279 23772685

[83] Zhou X Bailey-Bucktrout SL Jeker LT Penaranda C Martínez-Llordella M Ashby M Instability of the transcription factor Foxp3 leads to the generation of pathogenic memory T cells in vivo. Nat Immunol (2009) 10(9):1000–7. 10.1038/ni.1774 PMC2729804 19633673

[84] Rubtsov YP Niec RE Josefowicz S Li L Darce J Mathis D Stability of the regulatory T cell lineage in vivo. Science (2010) 329(5999):1667–71. 10.1126/science.1191996 PMC4262151 20929851

[85] Lu L Lan Q Li Z Zhou X Gu J Li Q Critical role of all-trans retinoic acid in stabilizing human natural regulatory T cells under inflammatory conditions. Proc Natl Acad Sci U S A (2014) 111(33):E3432–40. 10.1073/pnas.1408780111 PMC4143025 25099355

[86] Fu W Ergun A Lu T Hill JA Haxhinasto S Fassett MS A multiply redundant genetic switch “locks in” the transcriptional signature of regulatory T cells. Nat Immunol (2012) 13(10):972–80. 10.1038/ni.2420 PMC3698954 22961053

[87] Ehx G Fransolet G de Leval L D’Hondt S Lucas S Hannon M Azacytidine prevents experimental xenogeneic graft-versus-host disease without abrogating graft-versus-leukemia effects. Oncoimmunology (2017) 6(5):e1314425. 10.1080/2162402X.2017.1314425 28638744 PMC5467988

[88] Gregori S Roncarolo MG Engineered T Regulatory Type 1 Cells for Clinical Application. Front Immunol (2018) 9:233. 10.3389/fimmu.2018.00233 29497421 PMC5818395

[89] Locafaro G Andolfi G Russo F Cesana L Spinelli A Camisa B IL-10-Engineered Human CD4(+) Tr1 Cells Eliminate Myeloid Leukemia in an HLA Class I-Dependent Mechanism. Mol Ther (2017) 25(10):2254–69. 10.1016/j.ymthe.2017.06.029 PMC5628869 28807569

[90] Blazar BR MacDonald KPA Hill GR Immune regulatory cell infusion for graft-versus-host disease prevention and therapy. Blood (2018) 131(24):2651–60. 10.1182/blood-2017-11-785865 PMC6032895 29728401

[91] Negrin RS Immune regulation in hematopoietic cell transplantation. Bone Marrow Transplant (2019) 54(S2):765–8. 10.1038/s41409-019-0600-7 31431709

[92] Wang Z Liu X Cao F Bellanti JA Zhou J Zheng SG Prospects of the Use of Cell Therapy to Induce Immune Tolerance. Front Immunol (2020) 11:792. 10.3389/fimmu.2020.00792 32477335 PMC7235417

[93] Coman T Rossignol J D’Aveni M Fabiani B Dussiot M Rignault R Human CD4- invariant NKT lymphocytes regulate graft versus host disease. Oncoimmunology (2018) 7(11):e1470735. 10.1080/2162402X.2018.1470735 30377560 PMC6204997

[94] Schneidawind D Pierini A Alvarez M Pan Y Baker J Buechele C CD4+ invariant natural killer T cells protect from murine GVHD lethality through expansion of donor CD4+CD25+FoxP3+ regulatory T cells. Blood (2014) 124(22):3320–8. 10.1182/blood-2014-05-576017 PMC4239338 25293774

[95] Wu S-R Reddy P Tissue tolerance: a distinct concept to control acute GVHD severity. Blood (2017) 129(13):1747–52. 10.1182/blood-2016-09-740431 PMC5374290 28153825

[96] Wu S-R Reddy P Regulating Damage from Sterile Inflammation: A Tale of Two Tolerances. Trends Immunol (2017) 38(4):231–5. 10.1016/j.it.2017.02.005 PMC5384109 28268062

[97] Hayase E Hashimoto D Nakamura K Noizat C Ogasawara R Takahashi S R-Spondin1 expands Paneth cells and prevents dysbiosis induced by graft-versus-host disease. J Exp Med (2017) 214(12):3507–18. 10.1084/jem.20170418 PMC5716036 29066578

[98] Norona J Apostolova P Schmidt D Ihlemann R Reischmann N Taylor G Glucagon like peptide-2 for Intestinal stem cell and Paneth cell repair during graft-versus-host disease in mice and humans. Blood (2020) 136(12):1442–45. 10.1182/blood.2020005957 PMC7498363 32542357

[99] Vanclée A Lutgens LCHW Oving EBH Deutz NEP Gijbels MJJ Schouten HC Keratinocyte growth factor ameliorates acute graft-versus-host disease in a novel nonmyeloablative haploidentical transplantation model. Bone Marrow Transplant (2005) 36(10):907–15. 10.1038/sj.bmt.1705157 16151417

[100] Hanash AM Dudakov JA Hua G O’Connor MH Young LF Singer NV Interleukin-22 protects intestinal stem cells from immune-mediated tissue damage and regulates sensitivity to graft versus host disease. Immunity (2012) 37(2):339–50. 10.1016/j.immuni.2012.05.028 PMC3477611 22921121

[101] Dudakov JA Hanash AM van den Brink MRM Interleukin-22: Immunobiology and Pathology. Annu Rev Immunol (2015) 33(1):747–85. 10.1146/annurev-immunol-032414-112123 PMC4407497 25706098

[102] Köhler N Zeiser R Intestinal Microbiota Influence Immune Tolerance Post Allogeneic Hematopoietic Cell Transplantation and Intestinal GVHD. Front Immunol (2018) 9:3179. 10.3389/fimmu.2018.03179 30705680 PMC6344415

[103] Peled JU Gomes ALC Devlin SM Littmann ER Taur Y Sung AD Microbiota as Predictor of Mortality in Allogeneic Hematopoietic-Cell Transplantation. N Engl J Med (2020) 382(9):822–34. 10.1056/NEJMoa1900623 PMC7534690 32101664

[104] Holler E Butzhammer P Schmid K Hundsrucker C Koestler J Peter K Metagenomic analysis of the stool microbiome in patients receiving allogeneic stem cell transplantation: loss of diversity is associated with use of systemic antibiotics and more pronounced in gastrointestinal graft-versus-host disease. Biol Blood Marrow Transplant (2014) 20(5):640–5. 10.1016/j.bbmt.2014.01.030 PMC4973578 24492144

[105] Payen M Nicolis I Robin M Michonneau D Delannoye J Mayeur C Functional and phylogenetic alterations in gut microbiome are linked to graft-versus-host disease severity. Blood Adv (2020) 4(9):1824–32. 10.1182/bloodadvances.2020001531 PMC7218439 32353108

[106] Taur Y Jenq RR Perales M-A Littmann ER Morjaria S Ling L The effects of intestinal tract bacterial diversity on mortality following allogeneic hematopoietic stem cell transplantation. Blood (2014) 124(7):1174–82. 10.1182/blood-2014-02-554725 PMC4133489 24939656

[107] Legoff J Resche-Rigon M Bouquet J Robin M Naccache SN Mercier-Delarue S The eukaryotic gut virome in hematopoietic stem cell transplantation: new clues in enteric graft-versus-host disease. Nat Med (2017) 23(9):1080–5. 10.1038/nm.4380 28759053

[108] Jenq RR Taur Y Devlin SM Ponce DM Goldberg JD Ahr KF Intestinal Blautia Is Associated with Reduced Death from Graft-versus-Host Disease. Biol Blood Marrow Transplant (2015) 21(8):1373–83. 10.1016/j.bbmt.2015.04.016 PMC4516127 25977230

[109] Nishi K Kanda J Hishizawa M Kitano T Kondo T Yamashita K Impact of the Use and Type of Antibiotics on Acute Graft-versus-Host Disease. Biol Blood Marrow Transplant J Am Soc Blood Marrow Transplant (2018) 24(11):2178–83. 10.1016/j.bbmt.2018.06.031 30417828

[110] Shono Y Docampo MD Peled JU Perobelli SM Velardi E Tsai JJ Increased GVHD-related mortality with broad-spectrum antibiotic use after allogeneic hematopoietic stem cell transplantation in human patients and mice. Sci Transl Med (2016) 8(339):339ra71. 10.1126/scitranslmed.aaf2311 PMC4991773 27194729

[111] Simms-Waldrip TR Sunkersett G Coughlin LA Savani MR Arana C Kim J Antibiotic-Induced Depletion of Anti-inflammatory Clostridia Is Associated with the Development of Graft-versus-Host Disease in Pediatric Stem Cell Transplantation Patients. Biol Blood Marrow Transplant (2017) 23(5):820–9. 10.1016/j.bbmt.2017.02.004 28192251

[112] Riwes M Reddy P Short chain fatty acids: Postbiotics/metabolites and graft versus host disease colitis. Semin Hematol (2020) 57(1):1–6. 10.1053/j.seminhematol.2020.06.001 32690138 PMC9387163

[113] Riwes M Reddy P Microbial metabolites and graft versus host disease. Am J Transplant Off J Am Soc Transplant Am Soc Transpl Surg (2018) 18(1):23–9. 10.1111/ajt.14443 28742948

[114] Michonneau D Latis E Curis E Dubouchet L Ramamoorthy S Ingram B Metabolomics analysis of human acute graft-versus-host disease reveals changes in host and microbiota-derived metabolites. Nat Commun (2019) 10(1):5695. 10.1038/s41467-019-13498-3 31836702 PMC6910937

[115] Mathewson ND Jenq R Mathew AV Koenigsknecht M Hanash A Toubai T Gut microbiome-derived metabolites modulate intestinal epithelial cell damage and mitigate graft-versus-host disease. Nat Immunol (2016) 17(5):505–13. 10.1038/ni.3400 PMC4836986 26998764

[116] Servais S Beguin Y Delens L Ehx G Fransolet G Hannon M Novel approaches for preventing acute graft-versus-host disease after allogeneic hematopoietic stem cell transplantation. Expert Opin Invest Drugs (2016) 25(8):957–72. 10.1080/13543784.2016.1182498 27110922

[117] Penack O Marchetti M Ruutu T Aljurf M Bacigalupo A Bonifazi F Prophylaxis and management of graft versus host disease after stem-cell transplantation for haematological malignancies: updated consensus recommendations of the European Society for Blood and Marrow Transplantation. Lancet Haematol (2020) 7(2):e157–67. 10.1016/S2352-3026(19)30256-X 32004485

[118] Zeiser R Negrin RS Interleukin-2 receptor downstream events in regulatory T cells: Implications for the choice of immunosuppressive drug therapy. Cell Cycle (2008) 7(4):458–62. 10.4161/cc.7.4.5454 PMC2886808 18235249

[119] Cutler C Logan B Nakamura R Johnston L Choi S Porter D Tacrolimus/sirolimus vs tacrolimus/methotrexate as GVHD prophylaxis after matched, related donor allogeneic HCT. Blood (2014) 124(8):1372–7. 10.1182/blood-2014-04-567164 PMC4141519 24982504

[120] Sandmaier BM Kornblit B Storer BE Olesen G Maris MB Langston AA Addition of sirolimus to standard cyclosporine plus mycophenolate mofetil-based graft-versus-host disease prophylaxis for patients after unrelated non-myeloablative haemopoietic stem cell transplantation: a multicentre, randomised, phase 3 trial. Lancet Haematol (2019) 6(8):e409–18. 10.1016/S2352-3026(19)30088-2 PMC6686903 31248843

[121] Cutler C Stevenson K Kim HT Richardson P Ho VT Linden E Sirolimus is associated with veno-occlusive disease of the liver after myeloablative allogeneic stem cell transplantation. Blood (2008) 112(12):4425–31. 10.1182/blood-2008-07-169342 PMC2597119 18776081

[122] Walker I Panzarella T Couban S Couture F Devins G Elemary M Pretreatment with anti-thymocyte globulin versus no anti-thymocyte globulin in patients with haematological malignancies undergoing haemopoietic cell transplantation from unrelated donors: a randomised, controlled, open-label, phase 3, multicentre trial. Lancet Oncol (2016) 17(2):164–73. 10.1016/S1470-2045(15)00462-3 26723083

[123] Socie G Schmoor C Bethge WA Ottinger HD Stelljes M Zander AR Chronic graft-versus-host disease: long-term results from a randomized trial on graft-versus-host disease prophylaxis with or without anti-T-cell globulin ATG-Fresenius. Blood (2011) 117(23):6375–82. 10.1182/blood-2011-01-329821 21467544

[124] Kröger N Solano C Wolschke C Bandini G Patriarca F Pini M Antilymphocyte Globulin for Prevention of Chronic Graft-versus-Host Disease. N Engl J Med (2016) 374(1):43–53. 10.1056/NEJMoa1506002 26735993

[125] Soiffer RJ Kim HT McGuirk J Horwitz ME Johnston L Patnaik MM Prospective, Randomized, Double-Blind, Phase III Clinical Trial of Anti-T-Lymphocyte Globulin to Assess Impact on Chronic Graft-Versus-Host Disease-Free Survival in Patients Undergoing HLA-Matched Unrelated Myeloablative Hematopoietic Cell Transplantatio. J Clin Oncol (2017) 35(36):4003–11. 10.1200/JCO.2017.75.8177 PMC8462523 29040031

[126] Bonifazi F Solano C Wolschke C Sessa M Patriarca F Zallio F Acute GVHD prophylaxis plus ATLG after myeloablative allogeneic haemopoietic peripheral blood stem-cell transplantation from HLA-identical siblings in patients with acute myeloid leukaemia in remission: final results of quality of life and long-term outcome analysis of a phase 3 randomised study. Lancet Haematol (2019) 6(2):e89–99. 10.1016/S2352-3026(18)30214-X 30709437

[127] Soiffer RJ Lerademacher J Ho V Kan F Artz A Champlin RE Impact of immune modulation with anti-T-cell antibodies on the outcome of reduced-intensity allogeneic hematopoietic stem cell transplantation for hematologic malignancies. Blood (2011) 117(25):6963–70. 10.1182/blood-2011-01-332007 PMC3128486 21464372

[128] Servais S Menten-Dedoyart C Beguin Y Seidel L Gothot A Daulne C Impact of Pre-Transplant Anti-T Cell Globulin (ATG) on Immune Recovery after Myeloablative Allogeneic Peripheral Blood Stem Cell Transplantation. Boussiotis VA, editor. PLoS One (2015) 10(6):e0130026. 10.1371/journal.pone.0130026 26098781 PMC4476691

[129] Hannon M Beguin Y Ehx G Servais S Seidel L Graux C Immune Recovery after Allogeneic Hematopoietic Stem Cell Transplantation Following Flu-TBI versus TLI-ATG Conditioning. Clin Cancer Res (2015) 21(14):3131–9. 10.1158/1078-0432.CCR-14-3374 25779951

[130] Ali R Ramdial J Algaze S Beitinjaneh A The Role of Anti-Thymocyte Globulin or Alemtuzumab-Based Serotherapy in the Prophylaxis and Management of Graft-Versus-Host Disease. Biomedicines (2017) 5(4):67. 10.3390/biomedicines5040067 PMC5744091 29186076

[131] Baron F Mohty M Blaise D Socié G Labopin M Esteve J Anti-thymocyte globulin as graft-versus-host disease prevention in the setting of allogeneic peripheral blood stem cell transplantation: a review from the Acute Leukemia Working Party of the European Society for Blood and Marrow Transplantation. Haematologica (2017) 102(2):224–34. 10.3324/haematol.2016.148510 PMC5286931 27927772

[132] von dem Borne PA Starrenburg CWJI Halkes SJM Marijt WAE Fibbe WE Falkenburg JHF Reduced-intensity conditioning allogeneic stem cell transplantation with donor T-cell depletion using alemtuzumab added to the graft (‘Campath in the bag’). Curr Opin Oncol (2009) 21(1):S27–9. 10.1097/01.cco.0000357472.76337.0e 19561408

[133] Pasquini MC Devine S Mendizabal A Baden LR Wingard JR Lazarus HM Comparative outcomes of donor graft CD34+ selection and immune suppressive therapy as graft-versus-host disease prophylaxis for patients with acute myeloid leukemia in complete remission undergoing HLA-matched sibling allogeneic hematopoietic cell transplantation. J Clin Oncol (2012) 30(26):3194–201. 10.1200/JCO.2012.41.7071 PMC3434978 22869882

[134] Ho VT The history and future of T-cell depletion as graft-versus-host disease prophylaxis for allogeneic hematopoietic stem cell transplantation. Blood (2001) 98(12):3192–204. 10.1182/blood.V98.12.3192 11719354

[135] Nunes NS Kanakry CG Mechanisms of Graft-versus-Host Disease Prevention by Post-transplantation Cyclophosphamide: An Evolving Understanding. Front Immunol (2019) 10:2668. 10.3389/fimmu.2019.02668 31849930 PMC6895959

[136] Luznik L Bolanos-Meade J Zahurak M Chen AR Smith BD Brodsky R High-dose cyclophosphamide as single-agent, short-course prophylaxis of graft-versus-host disease. Blood (2010) 115(16):3224–30. 10.1182/blood-2009-11-251595 PMC2858487 20124511

[137] Luznik L O’Donnell PV Fuchs EJ Post-transplantation cyclophosphamide for tolerance induction in HLA-haploidentical bone marrow transplantation. Semin Oncol (2012) 39(6):683–93. 10.1053/j.seminoncol.2012.09.005 PMC3808078 23206845

[138] Luznik L O’Donnell PV Symons HJ Chen AR Leffell MS Zahurak M HLA-haploidentical bone marrow transplantation for hematologic malignancies using nonmyeloablative conditioning and high-dose, posttransplantation cyclophosphamide. Biol Blood Marrow Transplant (2008) 14(6):641–50. 10.1016/j.bbmt.2008.03.005 PMC2633246 18489989

[139] Elmariah H Fuchs EJ Post-transplantation cyclophosphamide to facilitate HLA-haploidentical hematopoietic cell transplantation: Mechanisms and results. Semin Hematol (2019) 56(3)::183–9. 10.1053/j.seminhematol.2018.09.002 PMC9229262 31202428

[140] Eto M Mayumi H Tomita Y Yoshikai Y Nishimura Y Nomoto K The requirement of intrathymic mixed chimerism and clonal deletion for a long-lasting skin allograft tolerance in cyclophosphamide-induced tolerance. Eur J Immunol (1990) 20(9):2005–13. 10.1002/eji.1830200919 2209702

[141] Eto M Mayumi H Tomita Y Yoshikai Y Nishimura Y Nomoto K Sequential mechanisms of cyclophosphamide-induced skin allograft tolerance including the intrathymic clonal deletion followed by late breakdown of the clonal deletion. J Immunol (1990) 145(5):1303–10. 2143514

[142] Kanakry CG Ganguly S Zahurak M Bolaños-Meade J Thoburn C Perkins B Aldehyde dehydrogenase expression drives human regulatory T cell resistance to posttransplantation cyclophosphamide. Sci Transl Med (2013) 5(211):211ra157. 10.1126/scitranslmed.3006960 PMC4155575 24225944

[143] Ganguly S Ross DB Panoskaltsis-Mortari A Kanakry CG Blazar BR Levy RB Donor CD4+ Foxp3+ regulatory T cells are necessary for posttransplantation cyclophosphamide-mediated protection against GVHD in mice. Blood (2014) 124(13):2131–41. 10.1182/blood-2013-10-525873 PMC4186542 25139358

[144] Baumeister SHC Rambaldi B Shapiro RM Romee R Key Aspects of the Immunobiology of Haploidentical Hematopoietic Cell Transplantation. Front Immunol (2020) 11:191. 10.3389/fimmu.2020.00191 32117310 PMC7033970

[145] El Fakih R Hashmi SK Ciurea SO Luznik L Gale RP Aljurf M Post-transplant cyclophosphamide use in matched HLA donors: a review of literature and future application. Bone Marrow Transplant (2019) 55(1):40–7. 10.1038/s41409-019-0547-8 31089284

[146] Bolaños-Meade J Reshef R Fraser R Fei M Abhyankar S Al-Kadhimi Z Three prophylaxis regimens (tacrolimus, mycophenolate mofetil, and cyclophosphamide; tacrolimus, methotrexate, and bortezomib; or tacrolimus, methotrexate, and maraviroc) versus tacrolimus and methotrexate for prevention of graft-versus-host disease with haemopoietic cell transplantation with reduced-intensity conditioning: a randomised phase 2 trial with a non-randomised contemporaneous control group (BMT CTN 1203). Lancet Haematol (2019) 6(3):e132–43. 10.1016/S2352-3026(18)30221-7 PMC6503965 30824040

[147] Jacoby E Varda-Bloom N Goldstein G Hutt D Churi C Vernitsky H Comparison of two cytoreductive regimens for αβ-T-cell-depleted haploidentical HSCT in pediatric malignancies: Improved engraftment and outcome with TBI-based regimen. Pediatr Blood Cancer (2018) 65(2):e26839. 10.1002/pbc.26839 28988422

[148] Bleakley M Heimfeld S Loeb KR Jones LA Chaney C Seropian S Outcomes of acute leukemia patients transplanted with naive T cell-depleted stem cell grafts. J Clin Invest (2015) 125(7):2677–89. 10.1172/JCI81229 PMC4563691 26053664

[149] Roy DC Lachance S Cohen S Delisle J Kiss T Sauvageau G Allodepleted T-cell immunotherapy after haploidentical haematopoietic stem cell transplantation without severe acute graft-versus-host disease (GVHD) in the absence of GVHD prophylaxis. Br J Haematol (2019) 186(5):754–66. 10.1111/bjh.15970 PMC6771482 31135970

[150] Koreth J Kim HT Lange PB Bindra B Reynolds CG Chammas MJ A Bortezomib-Based Regimen Offers Promising Survival and Graft-versus-Host Disease Prophylaxis in Myeloablative HLA-Mismatched and Unrelated Donor Transplantation: A Phase II Trial. Biol Blood Marrow Transplant (2015) 21(11):1907–13. 10.1016/j.bbmt.2015.05.027 PMC4604028 26055298

[151] Koreth J Kim HT Lange PB Poryanda SJ Reynolds CG Rai SC Bortezomib-based immunosuppression after reduced-intensity conditioning hematopoietic stem cell transplantation: randomized phase II results. Haematologica (2018) 103(3):522–30. 10.3324/haematol.2017.176859 PMC5830392 29326124

[152] Khandelwal P Yeh RF Yu L Lane A Dandoy CE El-Bietar J Graft Versus Host Disease Prophylaxis With Abatacept Reduces Severe Acute Graft Versus Host Disease in Allogeneic Hematopoietic Stem Cell Transplant for Beta Thalassemia Major with Busulfan, Fludarabine, and Thiotepa. Transplantation (2020). 10.1097/TP.0000000000003327. online ehaed of print. 32467478

[153] Koura DT Horan JT Langston AA Qayed M Mehta A Khoury HJ In vivo T cell costimulation blockade with abatacept for acute graft-versus-host disease prevention: a first-in-disease trial. Biol Blood Marrow Transplant (2013) 9(11):1638–49. 10.1016/j.bbmt.2013.09.003 24047754

[154] Jaiswal SR Bhakuni P Aiyer HM Soni M Bansal S Chakrabarti S CTLA4Ig in an Extended Schedule along with Sirolimus Improves Outcome with a Distinct Pattern of Immune Reconstitution Following Post-Transplantation Cyclophosphamide-Based Haploidentical Transplantation for Hemoglobinopathies. Biol Blood Marrow Transplant (2020) 26(8):1469–76. 10.1016/j.bbmt.2020.05.005 32428732

[155] Jaiswal SR Bhakuni P Zaman S Bansal S Bharadwaj P Bhargava S T cell costimulation blockade promotes transplantation tolerance in combination with sirolimus and post-transplantation cyclophosphamide for haploidentical transplantation in children with severe aplastic anemia. Transpl Immunol (2017) 43–44:54–9. 10.1016/j.trim.2017.07.004 28802588

[156] Jaiswal SR Bhakuni P Joy A Kaushal S Chakrabarti A Chakrabarti S CTLA4Ig Primed Donor Lymphocyte Infusion: A Novel Approach to Immunotherapy after Haploidentical Transplantation for Advanced Leukemia. Biol Blood Marrow Transplant (2019) 25(4):673–82. 10.1016/j.bbmt.2018.12.836 30610925

[157] Goodyear OC Dennis M Jilani NY Loke J Siddique S Ryan G Azacitidine augments expansion of regulatory T cells after allogeneic stem cell transplantation in patients with acute myeloid leukemia (AML). Blood (2012) 119(14):3361–9. 10.1182/blood-2011-09-377044 22234690

[158] Choi SW Braun T Henig I Gatza E Magenau J Parkin B Vorinostat plus tacrolimus/methotrexate to prevent GVHD after myeloablative conditioning, unrelated donor HCT. Blood (2017) 130(15):1760–7. 10.1182/blood-2017-06-790469 PMC5639486 28784598

[159] Choi SW Braun T Chang L Ferrara JLM Pawarode A Magenau JM Vorinostat plus tacrolimus and mycophenolate to prevent graft-versus-host disease after related-donor reduced-intensity conditioning allogeneic haemopoietic stem-cell transplantation: a phase 1/2 trial. Lancet Oncol (2014) 15(1):87–95. 10.1016/S1470-2045(13)70512-6 24295572 PMC4103793

[160] Khandelwal P Fukuda T Teusink-Cross A Kashuba ADM Lane A Mehta PA CCR5 inhibitor as novel acute graft versus host disease prophylaxis in children and young adults undergoing allogeneic stem cell transplant: results of the phase II study. Bone Marrow Transplant (2020) 55(8):1552–9. 10.1038/s41409-020-0888-3 32273585

[161] Chen Y-B Shah NN Renteria AS Cutler C Jansson J Akbari M Vedolizumab for prevention of graft-versus-host disease after allogeneic hematopoietic stem cell transplantation. Blood Adv (2019) 3(23):4136–46. 10.1182/bloodadvances.2019000893 PMC6963235 31821456

[162] Kennedy-Nasser AA Ku S Castillo-Caro P Hazrat Y Wu M-F Liu H Ultra low-dose IL-2 for GVHD prophylaxis after allogeneic hematopoietic stem cell transplantation mediates expansion of regulatory T cells without diminishing antiviral and antileukemic activity. Clin Cancer Res (2014) 20(8):2215–25. 10.1158/1078-0432.CCR-13-3205 PMC3989436 24573552

[163] Betts BC Pidala J Kim J Mishra A Nishihori T Perez L IL-2 promotes early Treg reconstitution after allogeneic hematopoietic cell transplantation. Haematologica (2017) 102(5):948–57. 10.3324/haematol.2016.153072 PMC5477614 28104702

[164] Elias S Rudensky AY Therapeutic use of regulatory T cells for graft-versus-host disease. Br J Haematol (2019) 187(1):25–38. 10.1111/bjh.16157 31418827 PMC8054701

[165] Zhao L Chen S Yang P Cao H Li L The role of mesenchymal stem cells in hematopoietic stem cell transplantation: prevention and treatment of graft-versus-host disease. Stem Cell Res Ther (2019) 10(1):182. 10.1186/s13287-019-1287-9 31227011 PMC6588914

[166] Chen Y-B Efebera YA Johnston L Ball ED Avigan D Lekakis LJ Increased Foxp3(+)Helios(+) Regulatory T Cells and Decreased Acute Graft-versus-Host Disease after Allogeneic Bone Marrow Transplantation in Patients Receiving Sirolimus and RGI-2001, an Activator of Invariant Natural Killer T Cells. Biol Blood Marrow Transplant (2017) 23(4):625–34. 10.1016/j.bbmt.2017.01.069 PMC5437739 28104514

[167] Yeh AC Brunner AM Spitzer TR Chen Y-B Coughlin E McAfee S Phase I Study of Urate Oxidase in the Reduction of Acute Graft-Versus-Host Disease after Myeloablative Allogeneic Stem Cell Transplantation. Biol Blood Marrow Transplant (2014) 20(5):730–4. 10.1016/j.bbmt.2014.02.003 24530972

[168] Jagasia MH Abonour R Long GD Bolwell BJ Laport GG Shore TB Palifermin for the reduction of acute GVHD: a randomized, double-blind, placebo-controlled trial. Bone Marrow Transplant (2012) 47(10):1350–5. 10.1038/bmt.2011.261 22327131

[169] Blazar BR Weisdorf DJ Defor T Goldman A Braun T Silver S Phase 1/2 randomized, placebo-control trial of palifermin to prevent graft-versus-host disease (GVHD) after allogeneic hematopoietic stem cell transplantation (HSCT). Blood (2006) 108(9):3216–22. 10.1182/blood-2006-04-017780 PMC1895527 16835378

[170] DeFilipp Z Hohmann E Jenq RR Chen Y-B Fecal Microbiota Transplantation: Restoring the Injured Microbiome after Allogeneic Hematopoietic Cell Transplantation. Biol Blood Marrow Transplant (2019) 25(1):e17–22. 10.1016/j.bbmt.2018.10.022 30408565

[171] DeFilipp Z Peled JU Li S Mahabamunuge J Dagher Z Slingerland AE Third-party fecal microbiota transplantation following allo-HCT reconstitutes microbiome diversity. Blood Adv (2018) 2(7):745–53. 10.1182/bloodadvances.2018017731 PMC5894265 29592876

[172] Gorshein E Wei C Ambrosy S Budney S Vivas J Shenkerman A Lactobacillus rhamnosus GG probiotic enteric regimen does not appreciably alter the gut microbiome or provide protection against GVHD after allogeneic hematopoietic stem cell transplantation. Clin Transplant (2017) 31(5):e12947. 10.1111/ctr.12947 28256022

[173] Al-Homsi AS Feng Y Duffner U Al Malki MM Goodyke A Cole K Bortezomib for the prevention and treatment of graft-versus-host disease after allogeneic hematopoietic stem cell transplantation. Exp Hematol (2016) 44(9):771–7. 10.1016/j.exphem.2016.05.005 27224851

[174] Rager A Frey N Goldstein SC Reshef R Hexner EO Loren A Inflammatory cytokine inhibition with combination daclizumab and infliximab for steroid-refractory acute GVHD. Bone Marrow Transplant (2011) 46(3):430–5. 10.1038/bmt.2010.117 PMC3010487 20498647

[175] Lee SJ Zahrieh D Agura E MacMillan ML Maziarz RT McCarthy PLJ Effect of up-front daclizumab when combined with steroids for the treatment of acute graft-versus-host disease: results of a randomized trial. Blood (2004) 104(5):1559–64. 10.1182/blood-2004-03-0854 15138163

[176] Antin JH Weisdorf D Neuberg D Nicklow R Clouthier S Lee SJ Interleukin-1 blockade does not prevent acute graft-versus-host disease: results of a randomized, double-blind, placebo-controlled trial of interleukin-1 receptor antagonist in allogeneic bone marrow transplantation. Blood (2002) 100(10):3479–82. 10.1182/blood-2002-03-0985 12393661

[177] Hamadani M Hofmeister CC Jansak B Phillips G Elder P Blum W Addition of infliximab to standard acute graft-versus-host disease prophylaxis following allogeneic peripheral blood cell transplantation. Biol Blood Marrow Transplant (2008) 14(7):783–9. 10.1016/j.bbmt.2008.04.006 PMC4100722 18541197

[178] Martelli MF Di Ianni M Ruggeri L Falzetti F Carotti A Terenzi A HLA-haploidentical transplantation with regulatory and conventional T-cell adoptive immunotherapy prevents acute leukemia relapse. Blood (2014) J124(4):638–44. 10.1182/blood-2014-03-564401 24923299

[179] Brunstein CG Miller JS Cao Q McKenna DH Hippen KL Curtsinger J Infusion of ex vivo expanded T regulatory cells in adults transplanted with umbilical cord blood: safety profile and detection kinetics. Blood (2011) 117(3):1061–70. 10.1182/blood-2010-07-293795 PMC3035067 20952687

[180] Di Ianni M Falzetti F Carotti A Terenzi A Castellino F Bonifacio E Tregs prevent GVHD and promote immune reconstitution in HLA-haploidentical transplantation. Blood (2011) 117(14):3921–8. 10.1182/blood-2010-10-311894elia 21292771

[181] Copsel S Wolf D Komanduri KV Levy RB The promise of CD4 ^+^ FoxP3 ^+^ regulatory T-cell manipulation *in vivo* : applications for allogeneic hematopoietic stem cell transplantation. Haematologica (2019) 104(7):1309–21. 10.3324/haematol.2018.198838 PMC6601084 31221786

[182] Kim B-S Nishikii H Baker J Pierini A Schneidawind D Pan Y Treatment with agonistic DR3 antibody results in expansion of donor Tregs and reduced graft-versus-host disease. Blood (2015) 126(4):546–57. 10.1182/blood-2015-04-637587 PMC4513255 26063163

[183] Malard F Labopin M Chevallier P Guillaume T Duquesne A Rialland F Larger number of invariant natural killer T cells in PBSC allografts correlates with improved GVHD-free and progression-free survival. Blood (2016) 127(14):1828–35. 10.1182/blood-2015-12-688739 26903546

[184] Amarnath S Foley JE Farthing DE Gress RE Laurence A Eckhaus MA Bone marrow-derived mesenchymal stromal cells harness purinergenic signaling to tolerize human Th1 cells in vivo. Stem Cells (2015) 33(4):1200–12. 10.1002/stem.1934 PMC4376558 25532725

[185] Bruck F Belle L Lechanteur C de Leval L Hannon M Dubois S Impact of bone marrow-derived mesenchymal stromal cells on experimental xenogeneic graft-versus-host disease. Cytotherapy (2013) 15(3):267–79. 10.1016/j.jcyt.2012.09.003 23265769

[186] Baron F Lechanteur C Willems E Bruck F Baudoux E Seidel L Cotransplantation of mesenchymal stem cells might prevent death from graft-versus-host disease (GVHD) without abrogating graft-versus-tumor effects after HLA-mismatched allogeneic transplantation following nonmyeloablative conditioning. Biol Blood Marrow Transplant (2010) 16(6):838–47. 10.1016/j.bbmt.2010.01.011 20109568

[187] Gao L Zhang Y Hu B Liu J Kong P Lou S Phase II Multicenter, Randomized, Double-Blind Controlled Study of Efficacy and Safety of Umbilical Cord-Derived Mesenchymal Stromal Cells in the Prophylaxis of Chronic Graft-Versus-Host Disease After HLA-Haploidentical Stem-Cell Transplantation. J Clin Oncol (2016) 34(24):2843–50. 10.1200/JCO.2015.65.3642 27400949

[188] Ning H Yang F Jiang M Hu L Feng K Zhang J The correlation between cotransplantation of mesenchymal stem cells and higher recurrence rate in hematologic malignancy patients: outcome of a pilot clinical study. Leukemia (2008) 22(3):593–9. 10.1038/sj.leu.2405090 18185520

[189] Magenau JM Goldstein SC Peltier D Soiffer RJ Braun T Pawarode A α(1)-Antitrypsin infusion for treatment of steroid-resistant acute graft-versus-host disease. Blood (2018) 131(12):1372–9. 10.1182/blood-2017-11-815746 PMC5865235 29437593

[190] Tawara I Sun Y Lewis EC Toubai T Evers R Nieves E Alpha-1-antitrypsin monotherapy reduces graft-versus-host disease after experimental allogeneic bone marrow transplantation. Proc Natl Acad Sci U S A (2012) 109(2):564–9. 10.1073/pnas.1117665109 PMC3258603 22203983

[191] Stein-Thoeringer CK Nichols KB Lazrak A Docampo MD Slingerland AE Slingerland JB Lactose drives Enterococcus expansion to promote graft-versus-host disease. Science (2019) 366(6469):1143–9. 10.1126/science.aax3760 PMC7003985 31780560

[192] Bouazzaoui A Huber E Dan A Al-Allaf FA Pfirstinger J Sprotte G Reduction of aGVHD using chicken antibodies directed against intestinal pathogens in a murine model. Blood (2017) 129(8):1052–5. 10.1182/blood-2016-06-722538 PMC5335808 28011676

[193] Gerbitz A Schultz M Wilke A Linde H-J Schölmerich J Andreesen R Probiotic effects on experimental graft-versus-host disease: let them eat yogurt. Blood (2004) 103(11):4365–7. 10.1182/blood-2003-11-3769 14962899

[194] Sadanand A Newland JG Bednarski JJ Safety of Probiotics Among High-Risk Pediatric Hematopoietic Stem Cell Transplant Recipients. Infect Dis Ther (2019) 8(2):301–6. 10.1007/s40121-019-0244-3 PMC6522555 30989592

[195] Schwartz M Gluck M Koon S Norovirus gastroenteritis after fecal microbiota transplantation for treatment of Clostridium difficile infection despite asymptomatic donors and lack of sick contacts. Am J Gastroenterol (2013) 108(8):1367. 10.1038/ajg.2013.164 23912408

[196] DeFilipp Z Bloom PP Torres Soto M Mansour MK Sater MRA Huntley MH Drug-Resistant E. coli Bacteremia Transmitted by Fecal Microbiota Transplant. N Engl J Med (2019) 381(21):2043–50. 10.1056/NEJMoa1910437 31665575

[197] Venkataraman A Sieber JR Schmidt AW Waldron C Theis KR Schmidt TM Variable responses of human microbiomes to dietary supplementation with resistant starch. Microbiome (2016) 4(1):33. 10.1186/s40168-016-0178-x 27357127 PMC4928258

